# Mutations of ribosomal protein genes induce overexpression of catalase in *Saccharomyces cerevisiae*

**DOI:** 10.1093/femsyr/foae005

**Published:** 2024-01-25

**Authors:** Ching-Hsiang Hsu, Ching-Yu Liu, Kai-Yin Lo

**Affiliations:** Department of Agricultural Chemistry National Taiwan University Agricultural Chemistry Building No. 2, Rm. 233 No. 1, Sec. 4, Roosevelt Rd. Taipei 10617, Taiwan; Department of Agricultural Chemistry National Taiwan University Agricultural Chemistry Building No. 2, Rm. 233 No. 1, Sec. 4, Roosevelt Rd. Taipei 10617, Taiwan; Department of Agricultural Chemistry National Taiwan University Agricultural Chemistry Building No. 2, Rm. 233 No. 1, Sec. 4, Roosevelt Rd. Taipei 10617, Taiwan

**Keywords:** ribosome, ribosomal proteins, reactive oxygen species, catalase, superoxide dismutase

## Abstract

Ribosome assembly defects result in ribosomopathies, primarily caused by inadequate protein synthesis and induced oxidative stress. This study aimed to investigate the link between deleting one ribosomal protein gene (RPG) paralog and oxidative stress response. Our results indicated that RPG mutants exhibited higher oxidant sensitivity than the wild type (WT). The concentrations of H_2_O_2_ were increased in the RPG mutants. Catalase and superoxide dismutase (SOD) activities were generally higher at the stationary phase, with catalase showing particularly elevated activity in the RPG mutants. While both catalase genes, *CTT1* and *CTA1*, consistently exhibited higher transcription in RPG mutants, Ctt1 primarily contributed to the increased catalase activity. Stress-response transcription factors Msn2, Msn4, and Hog1 played a role in regulating these processes. Previous studies have demonstrated that H_2_O_2_ can cleave 25S rRNA via the Fenton reaction, enhancing ribosomes’ ability to translate mRNAs associated with oxidative stress-related genes. The cleavage of 25S rRNA was consistently more pronounced, and the translation efficiency of *CTT1* and *CTA1* mRNAs was altered in RPG mutants. Our results provide evidence that the mutations in RPGs increase H_2_O_2_ levels *in vivo* and elevate catalase expression through both transcriptional and translational controls.

## Teaser

Mutations of ribosomal protein genes induce overexpression of catalase via transcriptional and translational controls in *Saccharomyces cerevisiae*.

## Introduction

The ribosome is a large complex composed of ribosomal RNAs and ribosomal proteins responsible for cellular translation. The entire process of ribosome synthesis, spanning from the nucleolus to the cytoplasm, involves the concerted efforts of hundreds of transacting factors and small nucleolar RNAs (snoRNAs). These factors play crucial roles in assisting with rRNA processing, the assembly of ribosomal proteins, and the overall reorganization of the ribosomal complex (Pena et al. 2017, Bassler and Hurt 2019, Klinge and Woolford 2019). Ribosome synthesis is a highly energy-intensive process that demands precise regulation, especially in response to variations in nutrient availability and environmental stresses (Matos-Perdomo and Machin 2019).

Defects in ribosome biogenesis or mutations in ribosomal proteins have been associated with a range of human congenital diseases, collectively referred to as ribosomopathies (Kang et al. 2021). Diamond–Blackfan anemia (DBA), for instance, is an inherited bone marrow failure syndrome characterized by symptoms such as anemia, congenital malformations, hypotrophy at birth, and growth retardation. In DBA, the involvement of 20 ribosomal protein genes (RPGs) has been identified (Da Costa et al. 2020). Another ribosomopathy is the Shwachman–Diamond syndrome (SDS), which is caused by mutations in the Shwachman–Bodian–Diamond syndrome (SBDS) gene. In yeast, the homolog of SBDS, Sdo1, has been identified as a critical release factor of the biogenesis factor Tif6 (Menne et al. 2007). This yeast model is consistent with observations in SDS, where ribosome assembly is defective (Wong et al. 2011). Additionally, a connection between ribosomal defects and cancer has been revealed. Mutations in ribosomal proteins and variations in ribosomal RNA (rRNA) copy numbers have been found in various types of tumors (Kampen et al. 2020, Pecoraro et al. 2021, Elhamamsy et al. 2022). Beyond impairing translation, these ribosomal mutations may alter the translation efficiency of oncoproteins, disrupt translation-independent functions of ribosomal proteins (Zhou et al. 2015), affect energy metabolism, and generate significant oxidative stress (Kampen et al. 2020). Consequently, targeting ribosome biogenesis has emerged as a potential therapeutic strategy (Jiao et al. 2023).

Proper ribosomal functioning is closely associated with maintaining cellular redox homeostasis (Kampen et al. 2020). Cells with ribosomal defects often exhibit increased levels of reactive oxygen species (ROS) (Ambekar et al. 2010, Turi et al. 2019). In pancreatic cancer cells, ribosomal protein Rpl10 (uL16) has been identified as a regulator of mitochondrial ROS levels (Yang et al. 2018). The *RPL10-R98S* mutation, which is associated with leukemia, enhances peroxisome activity, leading to elevated ROS levels (Kampen et al. 2019). ROS encompass byproducts generated during the incomplete reduction of oxygen, including superoxide anion (O_2_^−^), hydrogen peroxide (H_2_O_2_), and hydroxyl radical (HO^∙^). These unpaired ROS can potentially damage cellular macromolecules such as proteins, lipids, and DNA (Imlay 2003). ROS can act as signaling molecules at low concentrations that control intracellular ROS homeostasis (D’Autreaux and Toledano 2007). However, at higher concentrations, cells activate antioxidant mechanisms or may even initiate cell death via apoptosis (Perrone et al. 2008). Mitochondria, primarily responsible for powering cells, are a major intracellular source of ROS (Zorov et al. 2014).

In response to stress, several transcription factors play a crucial role in reprogramming gene expression in yeast (Jamieson 1998). Yap1, Yap2, and Gcn4 are bZip-type transcription factors with DNA-binding and dimerization domains (Fernandes et al. 1997). Yap1 mutants exhibit reduced activities of several antioxidant enzymes, including superoxide dismutase (SOD), glucose-6-phosphate dehydrogenase, and glutathione reductase (Schnell et al. 1992). Msn2 and Msn4, which share a 66% sequence homology and contain zinc finger binding motifs at the C-terminus (Estruch and Carlson 1993), are STRE (stress-responsive element)-binding proteins required for activating *CTT1, DDR2*, and *HSP12* (Schmitt and McEntee 1996). Mutants of Msn2 and Msn4 display heightened sensitivity to various stresses, including carbon source starvation, heat shock, osmotic stress, and oxidative stress (Estruch and Carlson 1993). Hog1 is a mitogen-activated protein kinase that responds to hyperosmolality (Brewster et al. 1993). In the presence of elevated levels of ROS, cleavage of 25S rRNA occurs at the ES7 region, resulting in preferential translation of mRNAs required for the oxidative stress response (Shedlovskiy et al. 2017). It is worth noting that this specific cleavage is an iron-dependent chemical reaction rather than an enzymatic mechanism (Zinskie et al. 2018).

In our study, we observed a significant increase in catalase activity among the RPG mutants during the stationary phase. Interestingly, this elevated catalase activity could be reduced by the addition of vitamin E but not by vitamin C. We identified the transcription factors Msn2, Msn4, and Hog1 as key regulators of this process. Furthermore, our analysis revealed a higher proportion of 25S rRNA cleavage in the RPG mutant strains, indicating an increase in oxidative stress. Additionally, we observed an enhanced translation efficiency of catalase genes, specifically *CTT1* and *CTA1*. These findings suggest that RPG mutations induce oxidative stress and activate the stress response through both transcriptional and translational regulatory mechanisms.

## Materials and methods

### Strains and media

All *Saccharomyces cerevisiae* strains used in this study are listed in Table S1 (Supporting Information). The strains derived from the Open Biosystems were validated by PCR to ensure the module had integrated at the correct positions before use (Longtine et al. 1998). To generate the deletion strains, the PCR products containing the KanMX (kanamycin resistance gene) (or clonNAT, nourseothricin sulfate resistance gene) and the flanking regions were transformed to the target strain, and the colonies that showed antibiotic resistance were picked up. The correct genome replacement of the gene with KanMX (or cloNAT) was examined with PCR (Longtine et al. 1998).

For the transformation process, yeast cells in the log growth phase were harvested and subsequently washed once with Li/TE (100 mM lithium acetate, 10 mM Tris, and 1 mM EDTA). The cells were then mixed with carrier DNA (single-strand DNA), purified PCR products, and PEG/Li/TE, followed by thorough mixing. The mixture was incubated for 30 min at 30°C and then for an additional 15 min at 42°C. Finally, the cells were spread onto the selective plates.

Unless otherwise indicated, all strains were grown at 30°C in a rich medium (yeast extract peptone) or synthetic dropout medium containing 2% glucose. The overnight culture was subcultured in the fresh medium and grown until the OD_600_ reached 0.3–0.5 (log phase), 1.0 (late log phase), or 4.0–6.0 (stationary phase).

### Growth test

The overnight cultures were normalized to OD 1 and subjected to 10-fold serial dilution. A volume of 5µl from each dilution was spotted on the various plates: yeast extract peptone dextrose (YPD) plates, YPD plates with 0.3 mM menadione or 5 mM H_2_O_2_, YPGal (yeast extract peptone galactose) plates or YPGal plates with 5 mM H_2_O_2_. Menadione is a superoxide-generating reagent that triggers cell oxidative stress (Kim et al. 2011).

### Western blotting

To detect the target proteins through western blotting, the proteins separated by SDS PAGE were transferred to a PVDF membrane (Bio-Rad) using a semidry transfer device (Bio-Rad). Membranes were incubated with TBST solution (Tris-buffered saline with 0.1% Tween® 20 detergent) containing primary antibodies. Anti-GFP (Thermo) and anti-TAP (Thermo) were purchased; anti-Act1 was derived from the Taiwan yeast resource center. anti-uL8 and anti-eS24 were generated in this lab. Protein signals were detected by Clarity^TM^ ECL Substrate (Bio-Rad). Images were acquired with MultiGel-21 (TopBio, Taiwan).

### RNA preparation and northern blotting

Total RNA was extracted from yeast cells using the hot phenol method (Collart and Oliviero 2001), and RNA was resolved in the formaldehyde agarose gel and transferred to a nitrocellulose membrane. The probes were labeled with a Biotin 3ʹend labeling kit (Thermo) and continually hybridized and detected with North2South^®^ Chemiluminescent hybridization and detection kit (Thermo). The probe sequence is listed in Table S2 (Supporting Information).

### Quantitative polymerase chain reaction (qPCR)

The cDNAs were synthesized with a High-Capacity cDNA Reverse Transcription Kit (Thermo). Real-time qPCR (RT-qPCR) was performed with a Power SYBR Green PCR Master Mix (Thermo) in a Real-Time PCR machine (Bio-Rad). *ACT1* was used as the internal control. Table S2 (Supporting Information) lists the primer sequences.

### Sucrose gradient analysis

The cell extracts were separated through a sucrose gradient to separate the translated and nontranslated mRNAs. Yeast cells were collected at different growth stages. Cycloheximide, a translation inhibitor, was added at the final 50 µg/ml concentration before cell collection to stabilize polysome structures. Polysome lysis buffer (10 mM Tris-HCl, pH 7.5, 100 mM KCl, 10 mM MgCl_2_, 6 mM β-mercaptoethanol, and 200 µg/ml cycloheximide) was used for the preparation of protein extracts. 10.5 OD_260_ units of protein extracts were loaded onto linear 7%–47% sucrose gradients and spun at 40 000 rpm in a rotor (SW40; Beckman) for 2.5 h. Gradient fractions were collected on a density gradient fraction system (Brandel), continuously measuring absorbance at 254 nm. A total of 10% TCA was added to each fraction for protein precipitation. The protein pellets were dissolved in 1x SDS sample buffer. Samples were resolved by SDS-PAGE and detected by western blotting. A volume of 500 µl from each fraction was extracted with Trizol (Thermo) to analyze the RNA levels. RNA was precipitated with ethanol and dissolved in the DEPC water. The RNA levels were measured with qPCR.

### ROS measurement

1.5 OD yeast cells of WT or RPG mutants were collected at different growth stages. The cells were incubated with 10 µM DCFDA (2′,7′-dichlorodihydrofluorescein diacetate, Sigma) for 30 min in the dark. DCFDA is a cell-permeable dye, which is converted to fluorescent after cleavage by intracellular esterases and oxidized by ROS. After washing once with PBS (phosphate buffered saline), cells were incubated in 2 M lithium acetate for 2 min and then in 0.01% SDS and 0.05% chloroform for 2 min. After centrifugation, the fluorescence intensity of the supernatant was measured with the plate reader (SpectraMax^®^ iD3, Molecular Device) (James et al. 2015). The same amount of cells under the same process without DCFDA staining was used for background fluorescence measurement.

To measure the H_2_O_2_ production rate, 0.5 OD cells were resuspended in the solution containing 50 µl 50 mM sodium phosphate buffer (pH = 7.4), 50 µl 50 µM Amplex red (Thermo), and 0.1 U/ml HRP (Sigma). The fluorescence intensity was measured at excitation/emission 571/585 nm in the plate reader at different time points. The H_2_O_2_ production amount in 1 min was calculated.

### Microscopy

Cells at the log and stationary phases were harvested and examined with fluorescence microscopy to image the cellular distribution of recombinant GFP (green fluorescent protein) protein. Fluorescence was visualized on a microscope (AxioScope A1; Zeiss) fitted with a Plan Apochromat 100 × 1.40 NA DIC objective and a digital microscopy camera (AxioCam MRm Rev. 3) controlled with AxioVision LE module Fluorescence Lite (Zeiss). Images were prepared using Photoshop (version 7.0; Adobe).

### Zymography

Zymography was used to detect the activities of catalase and SOD. Yeast cells at the log or stationary phase were harvested and lysed with glass beads in PBS. To test if antioxidants would quench the induction of catalase, *uL11a*Δ and *uL11b*Δ were cultured in the YPD medium with different concentrations of ascorbic acid (vitamin C) or α-tocopherol (vitamin E) to the stationary phase. To determine which catalase was induced in the RPG mutants, *ctt1*Δ and *cta1*Δ were included in the study.

After measuring the concentrations of proteins with Bradford assay, a fixed amount of proteins were loaded in 6% native PAGE gel and run at 80 V for 20 min and then 120 V for 100 min at 4°C. For catalase detection, the gel was incubated with 10 mM H_2_O_2_ for 15 min, and then 1% K_3_[Fe(CN)_6_] and 1% FeCl_3_ for 15 min. For SOD detection, the gel was incubated in 0.1% NBT (nitroblue tetrazolium) for 15 min. After washing with ddH_2_O once, the gel was incubated in the 0.1 M potassium phosphate buffer (pH = 7.8) containing 22 mM TEMED (N,N,N′,N′-Tetramethylethylenediamine) and 28 µM riboflavin for 15 min under the light. The WT sample was included in every gel as a control, and the relative ratios were compared to WT on the same gel. The intensity of bands was quantitated with Image J. Each assay was performed at least twice. The same preparations of samples were also resolved in SDS PAGE and detected with Coomassie blue staining as the loading controls.

### Flow cytometry

To measure mitochondrial membrane potentials, cells were stained with JC-1. JC-1 is a dye that could be accumulated in the mitochondria via the membrane potentials: cells appear orange or green in color when the membrane potentials accumulate higher or lower JC-1 amounts, respectively. 0.1 OD cells of WT and RPG mutants were collected at different growth stages and resuspended in 1 ml PBS containing 5 µM JC-1 for 30 min (Smiley et al. 1991). After washing twice, the cells were analyzed with flow cytometry (Cytomic FC 5000, Beckman Coulter). A total of 10 000 cells were counted for each test. The analyses were repeated twice independently.

### Statistical analysis

In qPCR analysis, the data were shown as mean ± SD, with three independent replicates (*n* = 3) for each condition. Statistical analysis was performed using analysis of variance (ANOVA), followed by a *post hoc* least significant difference (LSD) test for multiple comparisons. Significance levels were indicated: *P < .05, **P < .01, and ***P < .001. A two-tailed Student’s *T*-test was employed for pairwise comparisons between each mutant and the wild type (WT) at the same growth stage to assess statistical significance in these cases (*P < .05, **P < .01, and ***P < .001). The analyses were performed in the Microsoft Excel 2019.

## Results

### RPG mutants have a higher sensitivity to oxidative stress

*Saccharomyces cerevisiae* has a haploid genome, but among the 78 RPGs, 59 have two copies, designated as paralogs, such as uL11a and uL11b. Although these paralogous genes possess nearly identical amino acid sequences, differences in their 5′ untranslated regions (UTRs), introns, and 3′ UTR sequences lead to distinct behaviors (Komili et al. 2007). Several paralogs were selected in this study. Mutations in uL6a/6b, uL11a/11b, eL24a/24b, and eS24a/24b have previously been associated with DBA, whereas mutations in uL2a/2b, eL8a/8b, uL30a/30b, and eL43a/43b have not been reported (Danilova and Gazda 2015). As a result, a comparison of phenotypes between these two groups can offer valuable insights.

**Figure 1.**
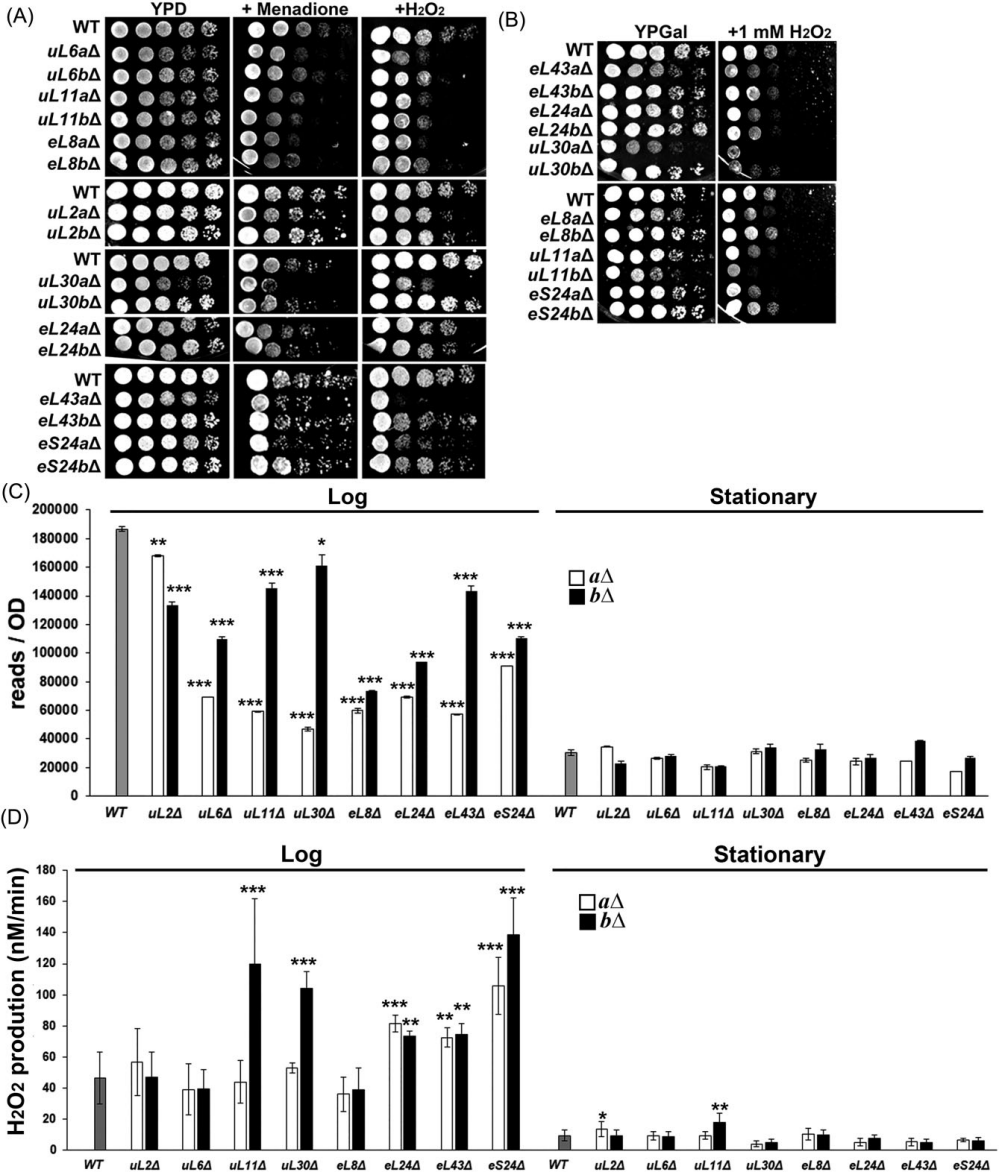
RPG mutants are more sensitive to oxidative stress. (A)–(C) Normalized cell cultures were serially diluted and spotted on the plates indicated in the figures at 30°C. (A) YPD and YPD with 0.3 mM menadione or 5 mM H_2_O_2_. (B) YPGal and YPGal with 1 mM H_2_O_2_. The growth test were done independently twice. (C) and (D) WT and the RPG mutants (*a*Δ was shown by a white bar and *b*Δ was shown by a black bar) at the log and stationary phases were collected. Total ROS levels were detected with DCFDA (C), and the H_2_O_2_ production rates were detected with Amplex Red (D). Each assay was done independently three times. The averages and standard deviations were shown. Each mutant was compared to WT at the same stage using the Student T test. *P < .05, **P < .01, and ***P < .001.

To assess the sensitivity of RPG mutants to oxidative stress, we conducted growth experiments on YPD plates containing varying concentrations of menadione and H_2_O_2_ (Fig. 1A). Most RPG mutants exhibited a growth rate similar to or slightly slower than the WT on YPD plates; however, their growth was significantly impaired in the presence of oxidants, indicating heightened sensitivity to oxidative stress. This trend persisted when we substituted the carbon source with another fermentative carbon source, galactose (Fig. 1B).

For further insight into cellular ROS levels, WT and RPG mutants were subjected to staining with DCFDA (Fig. 1C). Notably, the cells in the stationary phase displayed lower ROS, suggesting a correlation between ROS production and growth stages. Compared to WT, the mutants showed lower ROS at the log phase but similar levels at the stationary phase (Fig. 1C). To quantify H_2_O_2_ production rates, cells were also stained with Amplex red (Fig. 1D). Interestingly, many RPG mutants exhibited a higher rate of H_2_O_2_ generation during the log phase than WT (Fig. 1D).

### RPG mutants present higher catalase activity

Yeast employs nonenzymatic and enzymatic antioxidant defense systems to safeguard cells and maintain cellular redox balance. Among the major enzymatic systems responsible for ROS removal are SOD and catalase. We assessed SOD and catalase activities during the log and stationary growth phases to investigate whether RPG mutants exhibit elevated levels of these antioxidant enzymes.

**Figure 2.**
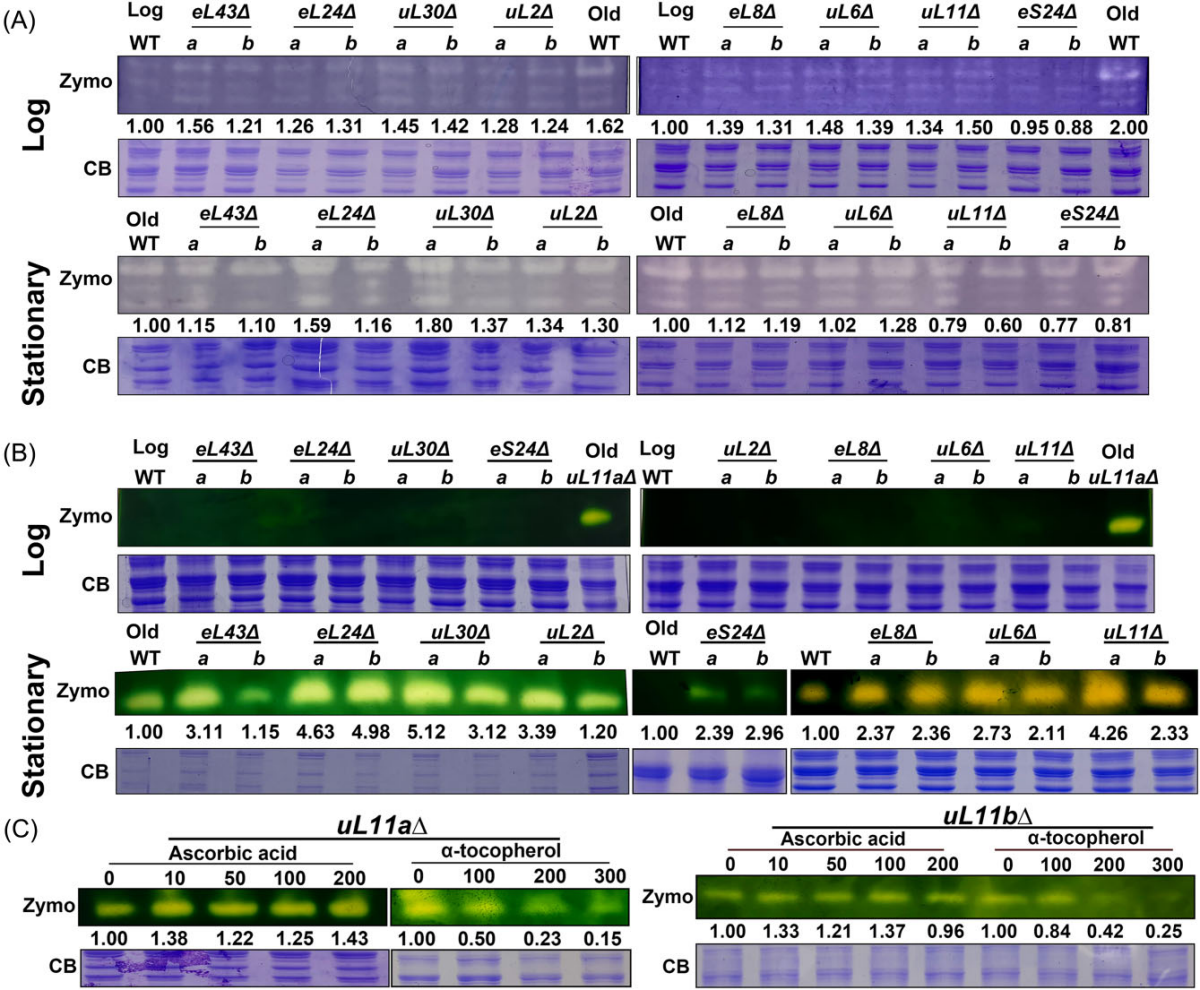
RPG mutants showed higher SOD and catalase activity, and treatment of α-tocopherol could reduce the catalase activity in the RPG mutant. (A) and (B) Cells in the log phase (top panel) and stationary phase (bottom panel) were collected, and the normalized whole cell lysates were prepared. The deletion of each paralog, *a*Δ or *b*Δ, was labeled as “a” or “b” on the figure. (A) 100 µg cell lysates were prepared from the log phase, and 30 µg cell lysates were prepared from the stationary phase. The SOD activity was analyzed with zymography. (B) 100 µg cell lysates were prepared from the log phase, and 50 µg cell lysates were prepared from the stationary phase. The catalase activity was analyzed with zymography. (C) *uL11a*Δ and *uL11b*Δ were cultured in the YPD medium with different concentrations of ascorbic acid or α-tocopherol to the stationary phase. The catalase activity was measured with zymography. The coomassie blue (CB) staining gels were included as the loading controls. Each assay was done independently at least two times. The relative ratios compared to WT were indicated in each figure.

In general, most RPG mutants showed a similar or slightly increased SOD activity to the WT (Fig. 2A). While neither WT nor the mutants in the log phase exhibited significant catalase activity, the catalase activity in most RPG mutants was 2–5 times higher than that of WT during the stationary growth phase (Fig. 2B). These results indicate that RPG mutations induce a more pronounced increase in catalase activity than SOD activity, consistent with our earlier observation of higher levels of H_2_O_2_ within the mutants (Fig. 1D).

Given the strong induction of catalase observed in most strains (Fig. 2B), we selected *uL11a*Δ and *uL11b*Δ for further examination. These mutants exhibited similar growth rates and sensitivity to oxidants (Fig. 1A and B) but displayed the most substantial differences in ROS and H_2_O_2_ levels (Fig. 1C and D). To assess whether the addition of antioxidants could suppress the overexpression of catalase, we introduced various concentrations of ascorbic acid (Vit C) and α–tocopherol (Vit E) into the cultures. Vit C, being water-soluble, and Vit E, an efficient lipid-soluble antioxidant that terminates lipid peroxidation (Nimse and Pal 2015), were chosen for this purpose. Notably, the addition of Vit E, but not Vit C, led to decreased catalase levels (Fig. 2C), suggesting that the increase in catalase expression was partially induced by ROS and lipid oxidation.

### RPG mutants show higher transcription and protein levels of the catalase

**Figure 3.**
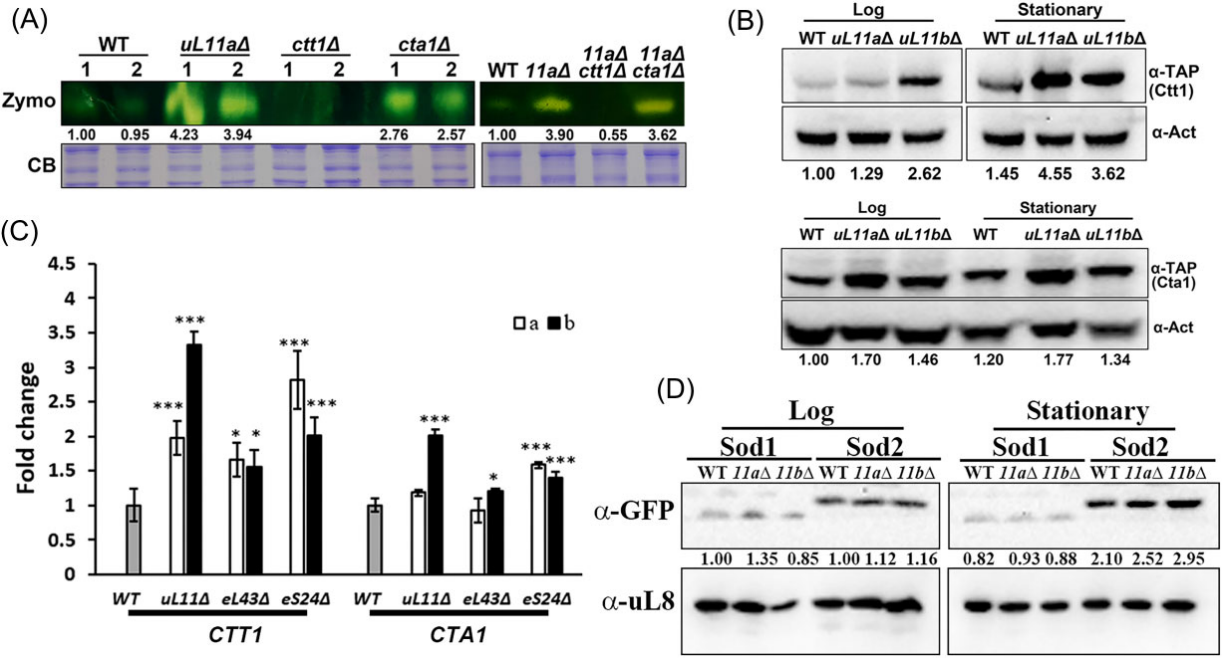
RPG mutants showed higher transcription and protein levels of the antioxidant enzymes. (A) The catalase activities in the mutant strains at the stationary phase were analyzed with zymography. The relative ratios compared to WT were indicated. The results of two replicates were shown in the left panel. (B) The protein levels of Ctt1 and Cta1 were analyzed with western blotting. The experiments were done twice. The quantification is indicated below the figure. (C) RNA was prepared from the WT and RPG mutants (*a*Δ was shown by a white bar and *b*Δ was shown by a black bar), and the transcription level of each gene was analyzed with qPCR. *ACT1* was used as the internal control. The data were shown as mean ± SD (*n* = 3) and analyzed with ANOVA followed by an LSD test. *P < .05; **P < .01; and ***P < .001. (D) The protein levels of Sod1 and Sod2 were analyzed with western blotting. The quantification is indicated below the figure.

There are two types of catalase in yeast: catalase T (Ctt1) in the cytosol and catalase A (Cta1) in peroxisomal and mitochondrial matrices (Cohen et al. 1985, Petrova et al. 2004). To determine which catalase was induced in the RPG mutants, the catalase zymography was examined in *ctt1*Δ, *cta1*Δ, and the combining mutants of *ctt1*Δ*uL11a*Δ and *cta1*Δ*uL11a*Δ. Remarkably, only the *ctt1*Δ strain displayed a significant loss of catalase activity (Fig. 3A). Subsequently, the protein quantities of Ctt1 and Cta1 were also checked in WT, *uL11a*Δ, and *uL11b*Δ strains. In the mutant strains, both proteins demonstrated elevated levels, with Ctt1 displaying a more pronounced increase during the stationary phase, whereas Cta1 exhibited consistent trends in both the log and stationary growth phases (Fig. 3B). The transcription levels of *CTT1* and *CTA1* were also checked: the mutants showed higher expressions than WT, and the increase of *CTT1* was more pronounced than *CTA1* at the log phase (Fig. 3C) but not at the stationary phase (Figure S2, Supporting Information).

The protein levels of two SODs were also examined. Sod1 is a Cu–Zn SOD localized in the cytosol and mitochondrial intermembrane space (Bermingham-McDonogh et al. 1988), and Sod2 is a mitochondrial MnSOD (van Loon et al. 1986). Sod1 levels remained constant in both WT and mutant strains at different growth phases, while Sod2 exhibited higher expression levels at the stationary phase, with slightly elevated levels in the mutants (Fig. 3D). These findings align with the insignificant increase in SOD activity observed in the RPG mutants.

The western blotting results of catalase or SOD were consistent with the zymography data (Fig. 2). Consequently, the RPG mutants displayed upregulated protein levels and activity of catalase, particularly evident during the stationary phase, with Ctt1 showing the most substantial changes among these enzymes.

### The transcription factors Msn2, Msn4, and Hog1 are responsible for the upregulation of catalase

**Figure 4.**
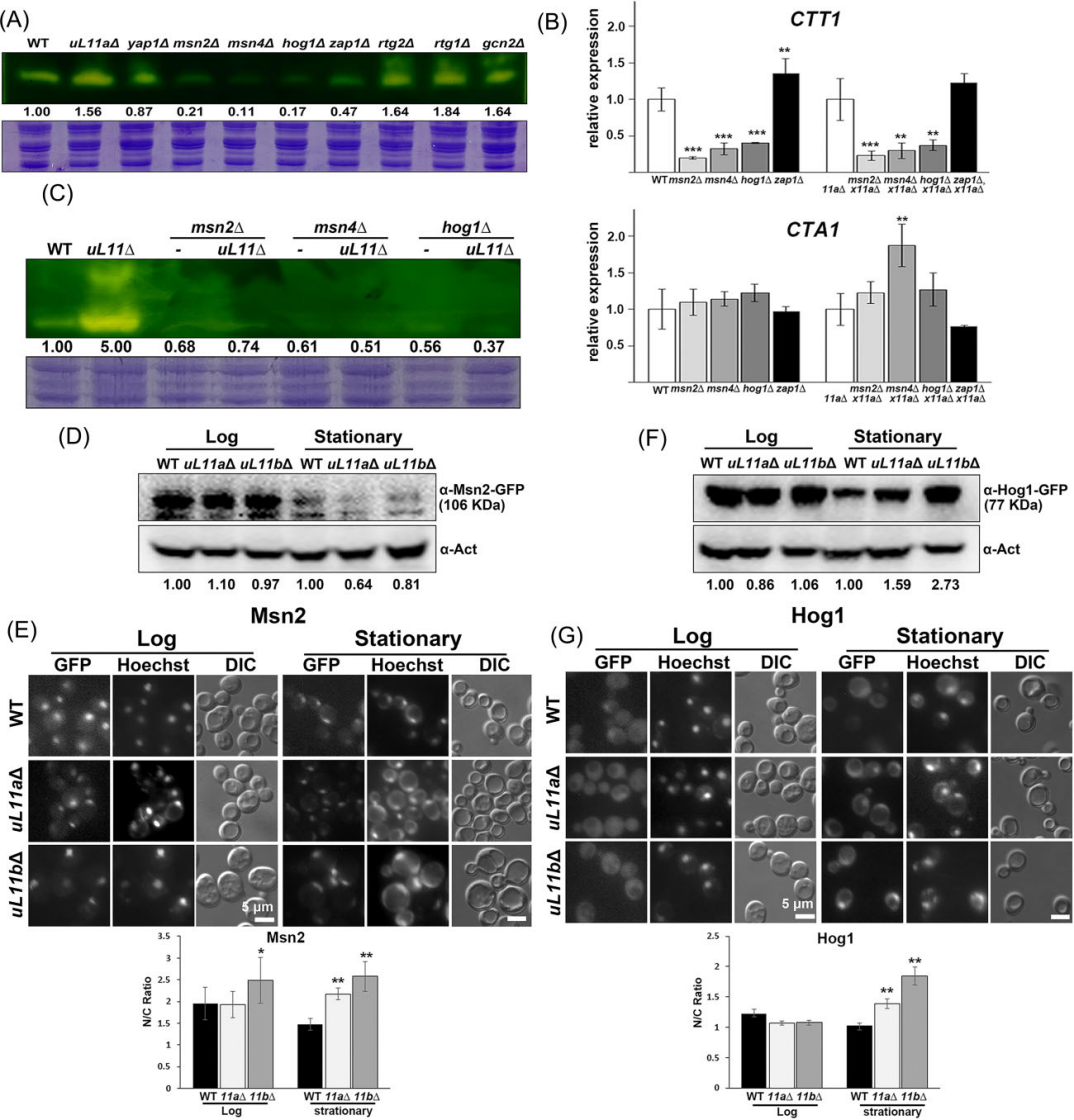
Msn2, Msn4, and Hog1 are responsible for the upregulation of catalase. (A) and (C) Cell extracts prepared from different strains at the stationary phase were analyzed with catalase zymography. The relative ratios compared to WT were indicated. (B) RNA was prepared from the cells at the log phase. The transcription levels were analyzed with qPCR. *ACT1* was used as the internal control. The data were shown as mean ± SD (*n* = 3) and analyzed with ANOVA followed by an LSD test. For each set, each stain was compared to WT or *uL11a*Δ. *P < .05, **P < .01, and ***P < .001. (D) and (F) The protein levels of Msn2–GFP and Hog1–GFP were analyzed with western blotting. The relative ratios compared to WT were shown. (E) and (G) The cellular localizations of Msn2–GFP and Hog1–GFP were examined in the cells at different growth stages. The N/C ratio was calculated from the intensity between the nucleus and cytoplasm from 20 cells. *uL11a*Δ or *uL11b*Δ was compared to WT at the same growth stage using the Student’s *T*-test. Ave ± SD. *P < .05 and **P < .01.

Several transcription factors involved in stress response were deleted to analyze which transcription factors are the major stress sensors of the RPG mutants, and the catalase activity was observed at the stationary phase. *msn2*Δ, *msn4*Δ, *hog1*Δ, and *zap1*Δ strains showed much lower catalase activity (Fig. 4A). The transcription levels of *CTT1* and *CTA1* were further checked in the *msn2*Δ, *msn4*Δ, *hog1*Δ, and *zap1*Δ mutants, as well as the double mutants crossed with *uL11a*Δ, to demonstrate the relationship. The RNA levels of *CTT1* were decreased in *msn2*Δ, *msn4*Δ, and *hog1*Δ but not in *zap1*Δ. In contrast, the *CTA1* RNA levels barely changed in these mutants (Fig. 4B). The catalase levels were also examined in these stains, and it was found that the absence of *MSN2, MSN4*, and *HOG1* decreased catalase activity in *uL11a*Δ (Fig. 4C).

Many transcription factors involved in stress response are induced by stress, resulting in an increase of the protein levels or relocalizing to the nucleus (Gorner et al. 1998, Westfall et al. 2004). The protein levels and cellular distributions of Msn2 and Hog1 were tracked in the WT and mutants at different growth stages. The Msn2–GFP (Rajvanshi et al. 2017, Mizuno and Irie 2021) and Hog1–GFP (Reiser et al. 1999) are regularly used to monitor protein status in response to stress or regulation. Therefore, Msn2–GFP and Hog1–GFP were examined in the WT, *uL11a*Δ, and *uL11b*Δ mutants.

The protein levels of Msn2 in the mutant strains were similar to those in the WT at the log phase and slightly decreased at the stationary phase (Fig. 4D). Msn2 was distributed in both the cytoplasm and nucleus in the WT and became more concentrated in the nucleus in the *uL11a*Δ and *uL11b*Δ mutants at the log and stationary phases (Fig. 4E). Hog1 levels remained unchanged in both the WT and mutants at the log phase but increased in the mutants at the stationary phase (Fig. 4F). Hog1 exhibited a stronger cytoplasmic intensity in the WT, and its nuclear intensity was higher in the mutant strains at the stationary phase (Fig. 4G). Thus, Msn2 and Hog1 adjusted their cellular localizations, and Hog1 even tuned up its protein abundances in response to the stress caused by the RPG mutants.

### Decrease in translation elongation triggers catalase overexpression

Ribosomal defects lead to an increase in ROS (Ambekar et al. 2010, Turi et al. 2019), and mitochondria are the principal places for ROS production. The mitochondria membrane potentials were measured in each mutant to investigate potential links between RPG mutants and mitochondrial status. Among the tested strains, only *uL6a*Δ, *uL6b*Δ, and *eS24a*Δ showed lower potential, while others displayed elevated potential (Figure S1, Supporting Information). However, every mutant strain exhibited higher catalase levels (Fig. 2B). Therefore, mitochondrial status might not be the key factor triggering catalase production.

**Figure 5.**
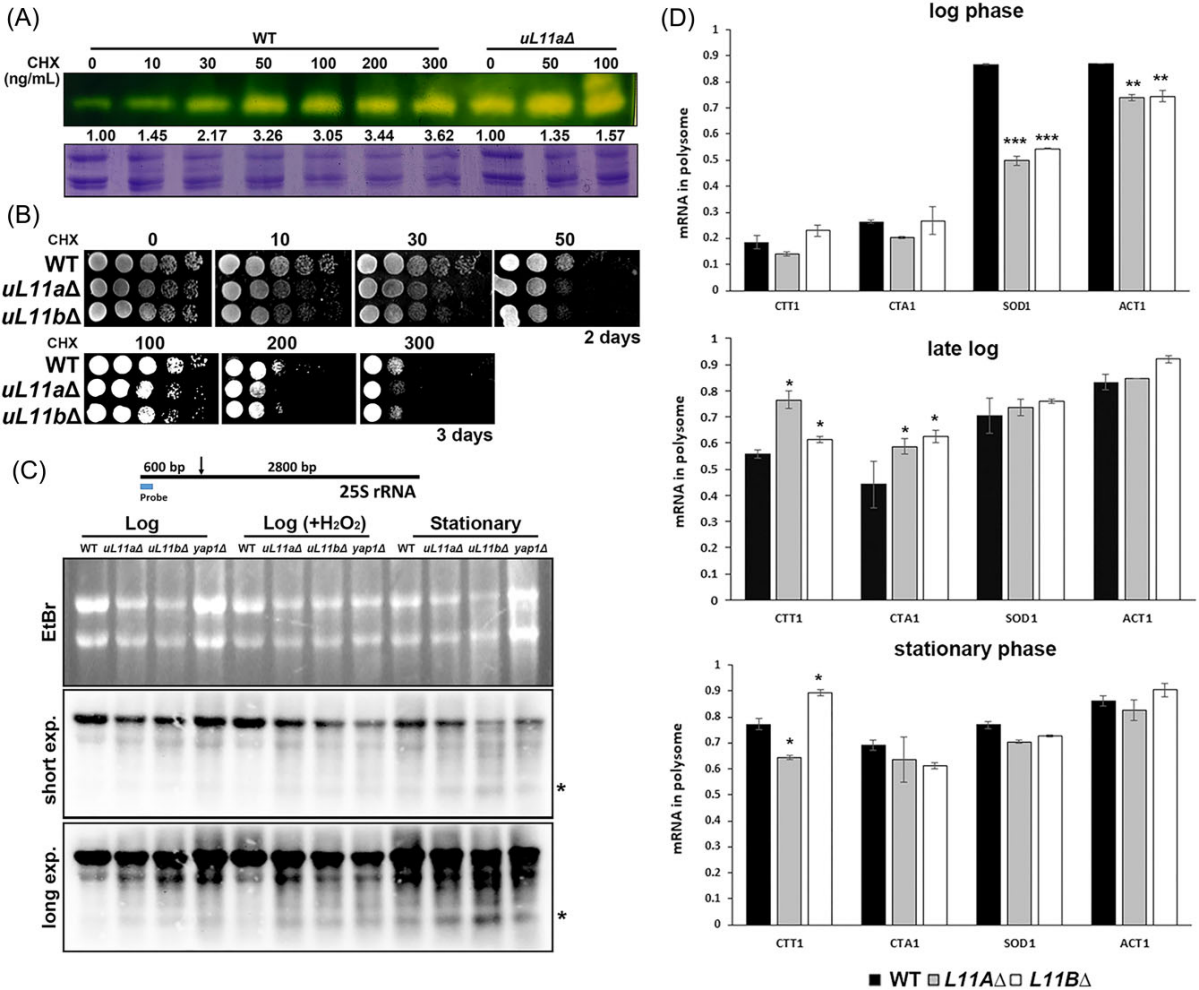
The overexpression of catalase in RPG mutants is also regulated at the translation level. (A) The cells were cultured with different concentrations of CHX to the stationary phase, and the catalase activity was analyzed with zymography. The relative ratios compared to WT were indicated. (B) The growth tests of strains on the plates containing different concentrations of CHX. (C) The position of the probe and the cleavage site at 25S rRNA were indicated. The cleaved 25S rRNAs were analyzed in the cells at log phase, log phase with the treatment of 0.25 mM H_2_O_2_ for 1 h, and stationary phase with northern blotting. The cleaved 25S rRNA band was indicated with an asterisk (*). (D) The fractions of mRNAs at the translation (the sum of the RNA levels at the 80S and polysome peaks) were analyzed at different growth stages with qPCR. Each mutant was compared to WT at the same stage using the Student’s *T*-test. *n* = 2 *P < .05; **P < .01; and ***P < .001.

Many important physiological pathways can be affected by ribosomal protein defects. Several mutant strains in the related pathways were selected to assess catalase levels (Figure S3A, Supporting Information). This information may be correlated with the primary factor triggering catalase production. The TOR complex is the primary sensor of nutrients and upregulates growth-related genes, including ribosome biogenesis and protein synthesis (Powers and Walter 1999). Deletion of Tor1, the component of TORC1, resulted in a slight increase in catalase activity. Gcn4 is the transcription activator that responds to amino acid starvation (Hinnebusch and Natarajan 2002), and the catalase activity did not increase in *gcn4*Δ. *TIF1* and *TIF4631* are one of the paralogous genes that code for translation initiation factors, specifically corresponding to eIF4A and eIF4G, respectively; *TIF3* is the coding gene of eIF4B (Altmann and Linder 2010). The catalase activity of *tif1*Δ, *tif3*Δ, and *tif4631*Δ was even lower than that of the WT (Figure S3A, Supporting Information). To investigate whether the translation defect caused the decrease in catalase levels, low amounts of cycloheximide (CHX), an inhibitor of translation elongation, were included in the culture and found to trigger catalase activity efficiently. The WT did not show growth defects at 30 ng/ml CHX (Fig. 5B), but this dosage triggered a 2-fold increase in catalase activity. Although the growth defects worsened in a dose-dependent manner, catalase activity plateaued at concentrations higher than 50 ng/ml CHX (Fig. 5A). *uL11a*Δ and *uL11b*Δ mutants showed higher growth defects toward CHX (Fig. 5B), and CHX even induced catalase activity in these mutants (Fig. 5A). Thus, translation elongation efficiency might be a critical factor triggering catalase overexpression.

### Overexpression of catalase in the RPG mutants is also regulated at the translational control level

The previous study showed that higher ROS would cleave 25S rRNA and change the mRNA preference of ribosomes (Shedlovskiy et al. 2017). Since H_2_O_2_ was higher in the RPG mutants (Fig. 1D), 25S rRNA might be cleaved in response to stress regulation. RNA was prepared from WT, *uL11a*Δ, and *uL11b*Δ, and the potential cleavage was observed with northern blotting. *yap1*Δ, a transcription factor for oxidative stress response, was included as a positive control as it has been shown to have 25S rRNA cleavage (Shedlovskiy et al. 2017). The cleavage was insignificant at the log phase and enhanced after H_2_O_2_ addition. At the stationary phase, compared to the WT, *uL11a*Δ, and *uL11b*Δ cells showed lower 25S rRNA and higher cleavage bands (Fig. 5C), which suggests the ROS levels were higher in these strains.

From the data above, disturbance in translation enhanced catalase expression (Fig. 5A), and the rRNA was cleaved in the RP mutants (Fig. 5C). Thus, translational control might be critical in adjusting the protein expressions related to the stress caused by RPG mutations. The translation profiles were analyzed in the WT and mutant strains at the log, late log, and stationary phases to dissect the dynamic responses. *SOD1* and *ACT1* (actin gene) were included for comparison with *CTT1* and *CTA1* to exclude the potential disturbance of translation from deletion of ribosomal protein. Cell extracts were fractioned through sucrose gradients, and the sedimentation positions of the 40S (frac. 3), 60S (frac. 4), 80S (frac. 5), and polysome (frac. 6–11) were checked with western blotting (Figure S3B, Supporting Information). RNA was extracted from each fraction and analyzed with qPCR. The fractions of nontranslated (frac. 1–4) and translated (frac. 5–11) were calculated. The RNA fractions in the polysome were compared between different growth stages of WT, *uL11a*Δ, and *uL11b*Δ strains (Fig. 5D). As the controls, the fractions of translated *ACT1* and *SOD1* were lower in the RPG mutants at the log phase, and no difference was observed between WT and RPG mutants at the late log or stationary phase. Meanwhile, only 20%–30% of *CTT1* and *CTA1* mRNAs were translated at the log phase, and this increased to 50%–80% at the late log phase as cells entered the stationary phase, with the RPG mutants exhibiting higher levels than the WT. Actively translated *CTA1* mRNAs achieved a plateau and no discrimination among the strains at the stationary phase. The changes in *CTT1* mRNAs were different: the translated fractions were increased to 80% and 90% in WT and *uL11b*Δ but decreased in *uL11a*Δ (Fig. 5D).

The data above suggest that RPG mutants showed different dynamics to trigger the translation of *CTT1* and *CTA1* mRNAs: the mutants translated the catalase genes at earlier stages than WT in response to the H_2_O_2_ levels in the cells. The upregulation of catalase genes in RPG mutants depends on transcriptional and translational controls to regulate oxidative homeostasis.

## Discussion

Many paralogs of RPGs were included in this study. The slow-growing RPG mutants showed higher sensitivity to oxidants in the growth test. However, many paralogs have comparable growth rates at normal conditions but behave with different sensitivity toward the oxidants (Fig. 1A and B). Although most paralogs have an identical amino acid sequence or only one or two amino acid differences, they may have unique functions. More and more studies have shown that ribosomes are created with heterogeneity. Different ribosomal protein paralogs, rRNA modifications, and post-translational modifications generate the “specialized ribosomes.” They demonstrate different preferences for specific mRNAs containing regulator elements, such as internal ribosome entry sites or upstream open reading frames, and behave as a regulator for cell homeostasis (Xue and Barna 2012, Norris et al. 2021).

We tried to correlate the catalase activity with the H_2_O_2_ generation rates. However, the connections were poor. For example, *eL43a*Δ and *eL43b*Δ showed similar rates (Fig. 1D), but *eL43a*Δ had higher catalase activity; *uL30b*Δ had higher H_2_O_2_ but *uL30a*Δ had higher catalase (Fig. 2B). The catalase activity correlated better with growth rates, while *eL43a*Δ and *uL30a*Δ had slower growth rates. Thus, the ROS level or H_2_O_2_ production is not the only factor to trigger catalase expression. In our study, we found Vit E but not Vit C could repress catalase overexpression (Fig. 2C). The difference might be due to their properties: Vit E is lipid soluble, and ViC is water soluble. While Vit E exerts antioxidant effects by scavenging lipid peroxyl radicals, it is not an efficient scavenger of ·OH and alkoxyl radicals (·OR) *in vivo* (Nimse and Pal 2015), which suggests that the signaling to induce catalase might be from the lipid sources.

The increase of catalase activity is growth stage-dependent: the activity was barely detected at the log phase, but it became obvious at the stationary stage (Fig. 2B). The previous study shows that peroxisome activity is enhanced in the *RPL10* mutant, resulting in elevated ROS levels (Kampen et al. 2019). Cta1, the catalase in the peroxisomal matrix, increased in RPG mutants but did not elevate from the log to the stationary phase (Fig. 3B). In contrast, Ctt1 increased at the stationary phase (Fig. 3B). When *CTT1* but not *CTA1* was deleted in *uL11a*Δ, the increase of catalase activity at the stationary phase disappeared (Fig. 3A right panel). Thus, the growth-dependent catalase activation in RPG mutants is mainly contributed by cytosolic catalase Ctt1. Deletion of *CTA1* but not *CTT1* induced the catalase levels, implying that the cells may upregulate the *CTT1* expression to compensate for the loss of *CTA1*, but not *vice versa* (Fig. 3A left panel).

The defects in RPG mutants may disturb other pathways besides translation, and ribosome biogenesis and translation are under many regulations. Thus, we included the mutants in other pathways in the assay: *tor1*Δ, the component of TORC1; *gcn4*Δ, the transcription activator in response to amino acid starvation; *tif1*Δ and *tif4631*Δ, one paralog of translation initiation factor eIF4A and eIF4G; *tif3*Δ, the coding gene of eIF4B (Altmann and Linder 2010). However, no catalase activity was significantly increased as RPG mutants in these strains (Figure S3A, Supporting Information). Thus, the elevated catalase activity might be unique to RPG mutations.

Out of the transcription factors we investigated, only the absence of Msn2, Msn4, or Hog1 led to a reduction in both catalase activity and transcription in the RPG mutants. These factors are known for recognizing stress-response elements (STRE), like AGGGG or GGGGA, particularly during stress conditions (Stewart-Ornstein et al. 2013). Notably, genes such as *CTT1, CTA1*, and *SOD1* also contain STRE elements (Rajvanshi et al. 2017). However, it is important to highlight that only the transcription levels of *CTT1* were adversely affected. This implies that the regulatory mechanisms governing these antioxidant enzymes may differ significantly.

Cells change protein synthesis in response to oxidative stress. Besides transcriptional control, translational control is also critical to maintaining homeostasis. H_2_O_2_ could regulate translation at the initiation, elongation, and termination stages (Grant 2011). In winter rye leaves, post-transcriptional control regulates the activation of catalase. The N7-methylation on the cap enhances the translation efficiency of catalase mRNA. Translation activation is induced by blue light and H_2_O_2_ (Schmidt et al. 2006). A previous study showed that higher ROS levels cleaved 25S rRNA and changed the mRNA preference of ribosomes in yeast (Shedlovskiy et al. 2017). In our data, *uL11b*Δ showed higher ROS levels (Fig. 1C); indeed, this strain showed a stronger cleavage band than WT and *uL11a*Δ (Fig. 5C). However, *uL11a*Δ showed similar ROS levels to WT (Fig. 1C) but still showed higher cleavage levels than WT (Fig. 5C). Another study further indicated that redox-active, ribosome-bound iron potentially promotes the Fenton reaction for rRNA cleavage (Zinskie et al. 2018). Thus, the mutant ribosome may have altered iron levels or bounding form, triggering the Fenton reaction. Our study found the translation efficiency of the *CTT1* and *CTA1* genes was enhanced starting from the late log phase. The translational preference might be from the cleavage of 25S rRNA in RPG mutant strains. Alternatively, the RPG deletion may change the ribosome selection toward certain mRNAs (Xue and Barna 2012). Or both factors contribute to the translational enhancement.

## Supplementary Material

foae005_Supplemental_File

## Data Availability

All data generated or analyzed during this study are included in this published article and its supplementary information files. The rest of the data generated in this study are available from corresponding authors on reasonable request.
