# Supplementary material for: Mutations of ribosomal protein genes induce overexpression of catalase in Saccharomyces cerevisiae
Source: FEMS Yeast Res. 2024 Jan 25;24:foae005. doi: 10.1093/femsyr/foae005 (PMC10855018; doi:10.1093/femsyr/foae005)
Supplement: foae005_Supplemental_File [file foae005_supplemental_file.pdf]

Table S1. Yeast strains

STRAIN	GENOTYPE	SOURCE
**BY4741**	MATa his3Δ1 leu2Δ0 met15Δ0 ura3Δ0	Open Biosystems
**KLY182**	MATa eL43aΔ::KanMX his3Δ1 leu2Δ0 met15Δ0 ura3Δ0	Open Biosystems
**KLY183**	MATa eL43bΔ::KanMX his3Δ1 leu2Δ0 met15Δ0 ura3Δ0	Open Biosystems
**KLY293**	MATa eL24aΔ::KanMX his3Δ1 leu2Δ0 met15Δ0 ura3Δ0	Open Biosystems
**KLY294**	MATa eL24bΔ::KanMX his3Δ1 leu2Δ0 met15Δ0 ura3Δ0	Open Biosystems
**KLY295**	MATa uL30aΔ::KanMX his3Δ1 leu2Δ0 met15Δ0 ura3Δ0	Open Biosystems
**KLY296**	MATa uL30bΔ::KanMX his3Δ1 leu2Δ0 met15Δ0 ura3Δ0	Open Biosystems
**KLY297**	MATa uL2aΔ::KanMX his3Δ1 leu2Δ0 met15Δ0 ura3Δ0	Open Biosystems
**KLY298**	MATa uL2bΔ::KanMX his3Δ1 leu2Δ0 met15Δ0 ura3Δ0	Open Biosystems
**KLY299**	MATa eL8aΔ::KanMX his3Δ1 leu2Δ0 met15Δ0 ura3Δ0	Open Biosystems
**KLY300**	MATa eL8bΔ::KanMX his3Δ1 leu2Δ0 met15Δ0 ura3Δ0	Open Biosystems
**KLY301**	MATa uL6aΔ::KanMX his3Δ1 leu2Δ0 met15Δ0 ura3Δ0	Open Biosystems
**KLY302**	MATa uL6bΔ::KanMX his3Δ1 leu2Δ0 met15Δ0 ura3Δ0	Open Biosystems
**KLY303**	MATa uL11aΔ::KanMX his3Δ1 leu2Δ0 met15Δ0 ura3Δ0	Open Biosystems
**KLY304**	MATa uL11bΔ::KanMX his3Δ1 leu2Δ0 met15Δ0 ura3Δ0	Open Biosystems
**KLY307**	MATa eS24aΔ::KanMX his3Δ1 leu2Δ0 met15Δ0 ura3Δ0	Open Biosystems
**KLY308**	MATa eS24bΔ::KanMX his3Δ1 leu2Δ0 met15Δ0 ura3Δ0	Open Biosystems
**KLY398**	MATa tor1Δ::KanMX his3Δ1 leu2Δ0 met15Δ0 ura3Δ0	Open Biosystems
**KLY425**	MATa yap1Δ::KanMX his3Δ1 leu2Δ0 met15Δ0 ura3Δ0	Open Biosystems
**KLY427**	MATa msn2Δ::KanMX his3Δ1 leu2Δ0 met15Δ0 ura3Δ0	Open Biosystems
**KLY428**	MATa msn4Δ::KanMX his3Δ1 leu2Δ0 met15Δ0 ura3Δ0	Open Biosystems
**KLY429**	MATa hog1Δ::KanMX his3Δ1 leu2Δ0 met15Δ0 ura3Δ0	Open Biosystems
**KLY430**	MATa zap1Δ::KanMX his3Δ1 leu2Δ0 met15Δ0 ura3Δ0	Open Biosystems
**KLY469**	MATa ctt1Δ::KanMX his3Δ1 leu2Δ0 met15Δ0 ura3Δ0	Open Biosystems
**KLY472**	MATa rtg2Δ::KanMX his3Δ1 leu2Δ0 met15Δ0 ura3Δ0	Open Biosystems
**KLY473**	MATa cta1Δ::KanMX his3Δ1 leu2Δ0 met15Δ0 ura3Δ0	Open Biosystems
**KLY474**	MATa rtg1Δ::KanMX his3Δ1 leu2Δ0 met15Δ0 ura3Δ0	Open Biosystems
**KLY785**	MATa gcn2Δ::KanMX his3Δ1 leu2Δ0 met15Δ0 ura3Δ0	Open Biosystems
**KLY786**	MATa gcn4Δ::KanMX his3Δ1 leu2Δ0 met15Δ0 ura3Δ0	Open Biosystems
**KLY846**	MATa tif1Δ::KanMX his3Δ1 leu2Δ0 met15Δ0 ura3Δ0	Open Biosystems
**KLY849**	MATa tif3Δ::KanMX his3Δ1 leu2Δ0 met15Δ0 ura3Δ0	Open Biosystems
**KLY859**	MATa tif4631Δ::KanMX his3Δ1 leu2Δ0 met15Δ0 ura3Δ0	Open Biosystems
**KLY1048**	MATa MSN2-GFP::HIS3 his3Δ1 leu2Δ0 met15Δ0 ura3Δ0	Open Biosystems
**KLY1328**	MATa CTT1-TAP::HIS3 his3Δ1 leu2Δ0 met15Δ0 ura3Δ0	Open Biosystems
**KLY1330**	MATa CTA1-TAP::HIS3 his3Δ1 leu2Δ0 met15Δ0 ura3Δ0	Open Biosystems
**KLY1438**	MATa uL11aΔ::CloNAT his3Δ1 leu2Δ0 met15Δ0 ura3Δ0	This study
**KLY1450**	MATa uL11aΔ::CloNAT msn2Δ::KanMX his3Δ1 leu2Δ0 met15Δ0 ura3Δ0	This study
**KLY1452**	MATa uL11aΔ::CloNAT msn4Δ::KanMX his3Δ1 leu2Δ0 met15Δ0 ura3Δ0	This study
**KLY1454**	MATa uL11aΔ::CloNAT hog1Δ::KanMX his3Δ1 leu2Δ0 met15Δ0 ura3Δ0	This study
**KLY1457**	MATa uL11aΔ::CloNAT ctt1Δ::KanMX his3Δ1 leu2Δ0 met15Δ0 ura3Δ0	This study

**KLY1459**	*MAT*a *uL11a*Δ::*CloNAT zap1*Δ::*KanMX his3*Δ1 *leu2*Δ0 *met15*Δ0 *ura3*Δ0	This study
**KLY1461**	*MAT*a *uL11a*Δ::*CloNAT cta1*Δ::*KanMX his3*Δ1 *leu2*Δ0 *met15*Δ0 *ura3*Δ0	This study
**KLY1467**	*MAT*a *HOG1-GFP::HIS3 leu2*Δ0 *met15*Δ0 *ura3Δ*0	Open Biosystems
**KLY1468**	*MAT*a *MSN2-GFP::HIS3 uL11a*Δ::*KanMX his3*Δ1 *leu2*Δ0 *met15*Δ0 *ura3Δ*0	This study
**KLY1470**	*MAT*a *HOG1-GFP::HIS3 uL11a*Δ::*KanMX his3*Δ1 *leu2*Δ0 *met15*Δ0 *ura3Δ*0	This study
**KLY1479**	*MAT*a *CTT1-TAP::HIS3 uL11a*Δ::*KanMX his3*Δ1 *leu2*Δ0 *met15*Δ0 *ura3Δ*0	This study
**KLY1481**	*MAT*a *CTT1-TAP::HIS3 uL11b*Δ::*KanMX his3*Δ1 *leu2*Δ0 *met15*Δ0 *ura3Δ*0	This study
**KLY1486**	*MAT*a *CTA1-TAP::HIS3 uL11a*Δ::*KanMX his3*Δ1 *leu2*Δ0 *met15*Δ0 *ura3Δ*0	This study
**KLY1488**	*MAT*a *CTA1-TAP::HIS3 uL11b*Δ::*KanMX his3*Δ1 *leu2*Δ0 *met15*Δ0 *ura3Δ*0	This study
**KLY1492**	*MAT*a *SOD1-GFP::HIS3 his3*Δ1 *leu2*Δ0 *met15*Δ0 *ura3Δ*0	This study
**KLY1493**	*MAT*a *SOD2-GFP::HIS3 his3*Δ1 *leu2*Δ0 *met15*Δ0 *ura3Δ*0	This study
**KLY1522**	*MAT*a *SOD1-GFP::HIS3 uL11a*Δ::*KanMX his3*Δ1 *leu2*Δ0 *met15*Δ0 *ura3Δ*0	This study
**KLY1524**	*MAT*a *SOD1-GFP::HIS3 uL11b*Δ:: *KanMX his3*Δ1 *leu2*Δ0 *met15*Δ0 *ura3Δ*0	This study
**KLY1526**	*MAT*a *SOD2-GFP::HIS3 uL11a*Δ::*KanMX his3*Δ1 *leu2*Δ0 *met15*Δ0 *ura3Δ*0	This study
**KLY1528**	*MAT*a *SOD2-GFP::HIS3 uL11b*Δ:: *KanMX his3*Δ1 *leu2*Δ0 *met15*Δ0 *ura3Δ*0	This study

Table S2. Primers and probes used in this study

qPCR	Primers Sequence (F: Forward, R: Reverse 5'→3')		
ACT1	F:	AGAGTTGCCCCAGAAGAACA	
	R:	GGCTTGGATGGAAACGTAGA	
CTT1	F:	AGAGAGTTACGCAATACTTTGG	
	R:	CCTTCAAGGTCAACAGGTTC	
CTA1	F:	CCAACAGGACAGACCCATTC	
	R:	TTACCCAAAACGCGGTAGAG	
SOD1	F:	TGTAGGCAGAAGCGTCGTTA	
	R:	ACCGGCATTACCAGTCTTCA	
probe	sequence		Ref.
25S RNA	TCCTACCTGATTTGAGGTCAAAC		(Shedlovskiy et al., 2017a)

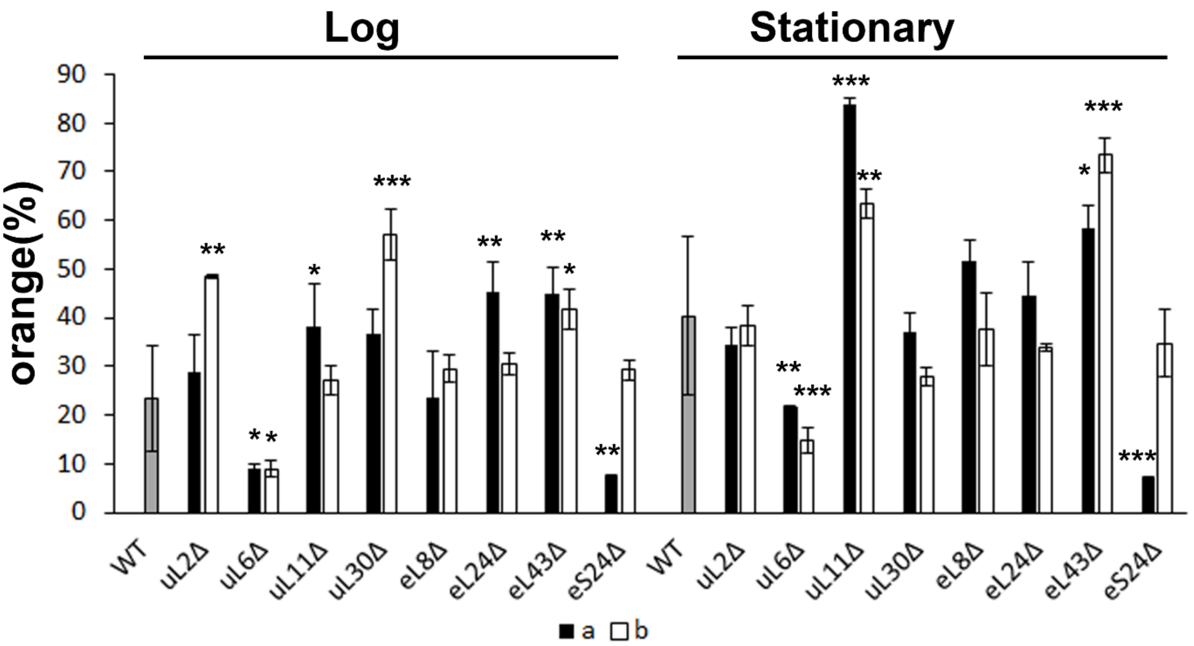

Fig S1. RPG mutants showed abnormal mitochondrial membrane potential

WT and RPG mutants (*a*Δ was shown by a black bar and *b*Δ was shown by a white bar) at different stages were stained with 5 μM JC-1 and analyzed with flow cytometry (Cytomic FC 5000, Beckman Coulter). 10000 cells were could for each test. Each mutant was compared to WT at the same stage using the Student T test. n=2. *p < 0.05, **p < 0.01, ***p < 0.001

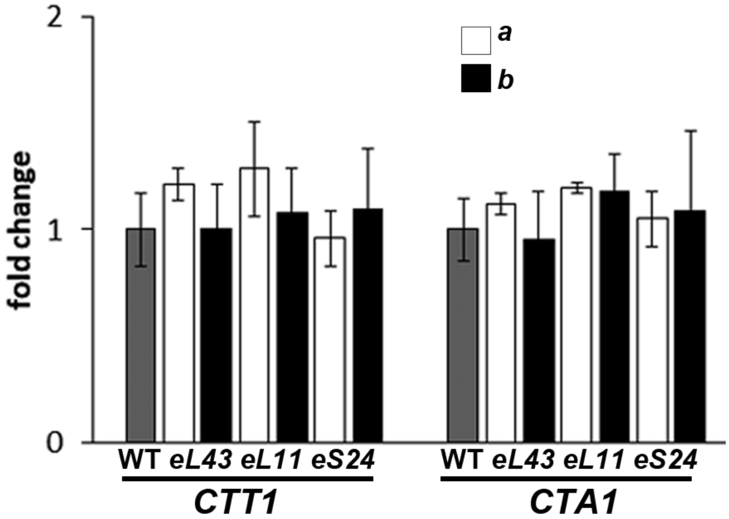

Fig S2. The transcription levels of *CTT1* and *CTA1* were similar among WT and RPG mutant strains at the stationary phase

RNA was prepared from the WT and RPG mutants (*a*Δ was shown by a black bar and *b*Δ was shown by a white bar) at the stationary phase, and the transcription level of each gene was analyzed with qPCR. n=3.

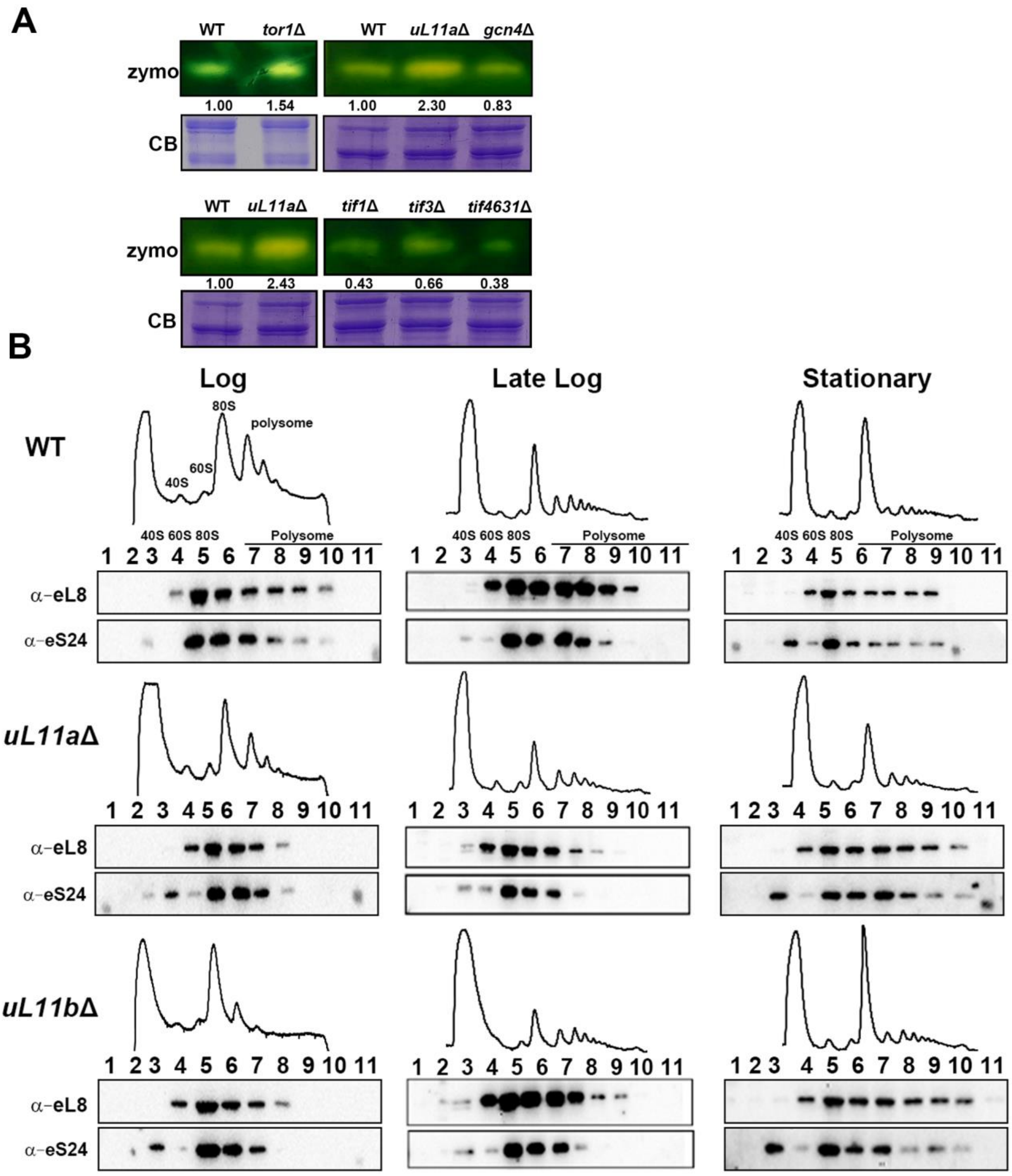

Fig S3. The catalase activities were examined in other mutants and the polysome profiles of WT and RPG mutants at different growth stages

(A) Cell extracts prepared from different strains at the stationary phase were analyzed with catalase zymography. The coomassie blue (CB) staining gels were included as the loading controls. Each assay was done independently at least two times. (B)The polysome profiles and the distributions of ribosomal subunits were tracked with western blotting. The positions of 40S and 60S subunits were probed with anti-eS24 and anti-eL8.

Figure 2A
Top panel

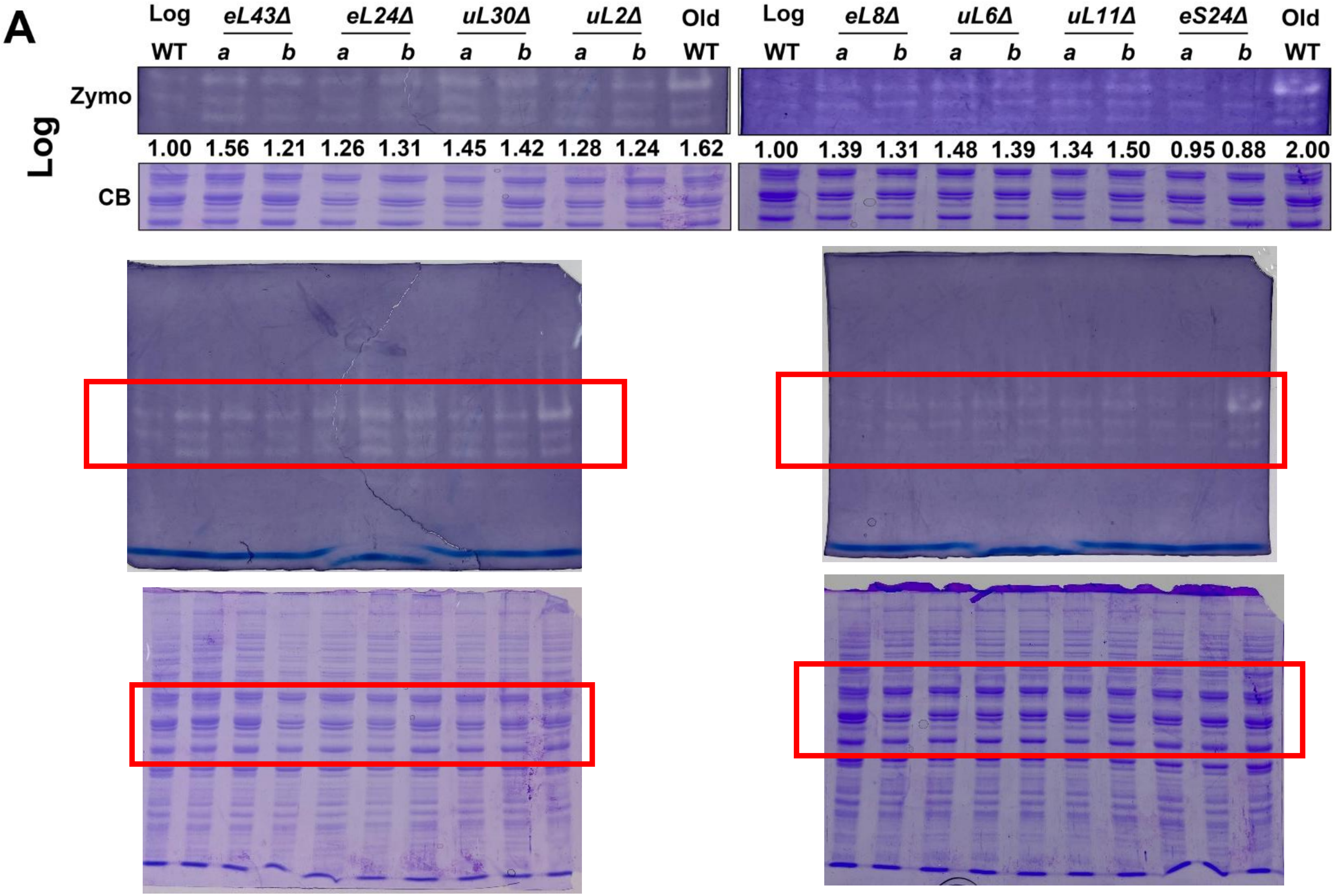

Figure 2A
Bottom panel

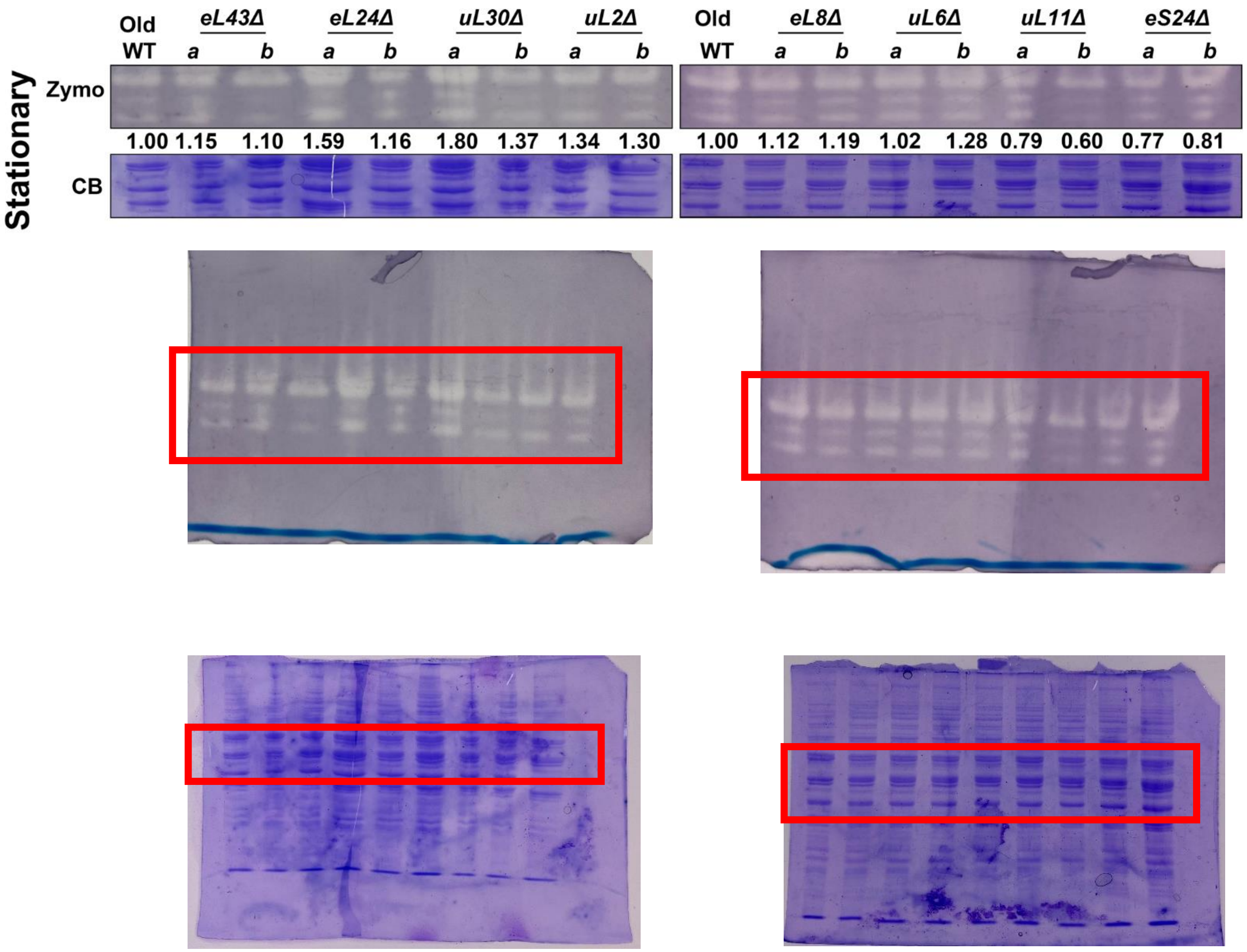

Figure 2B
Top panel

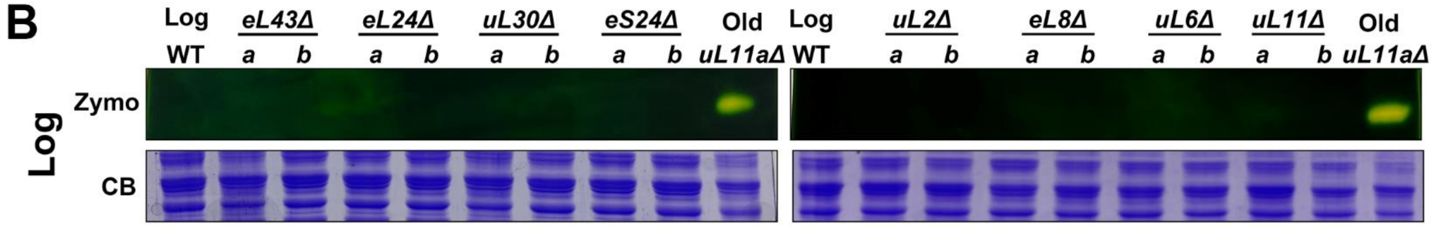

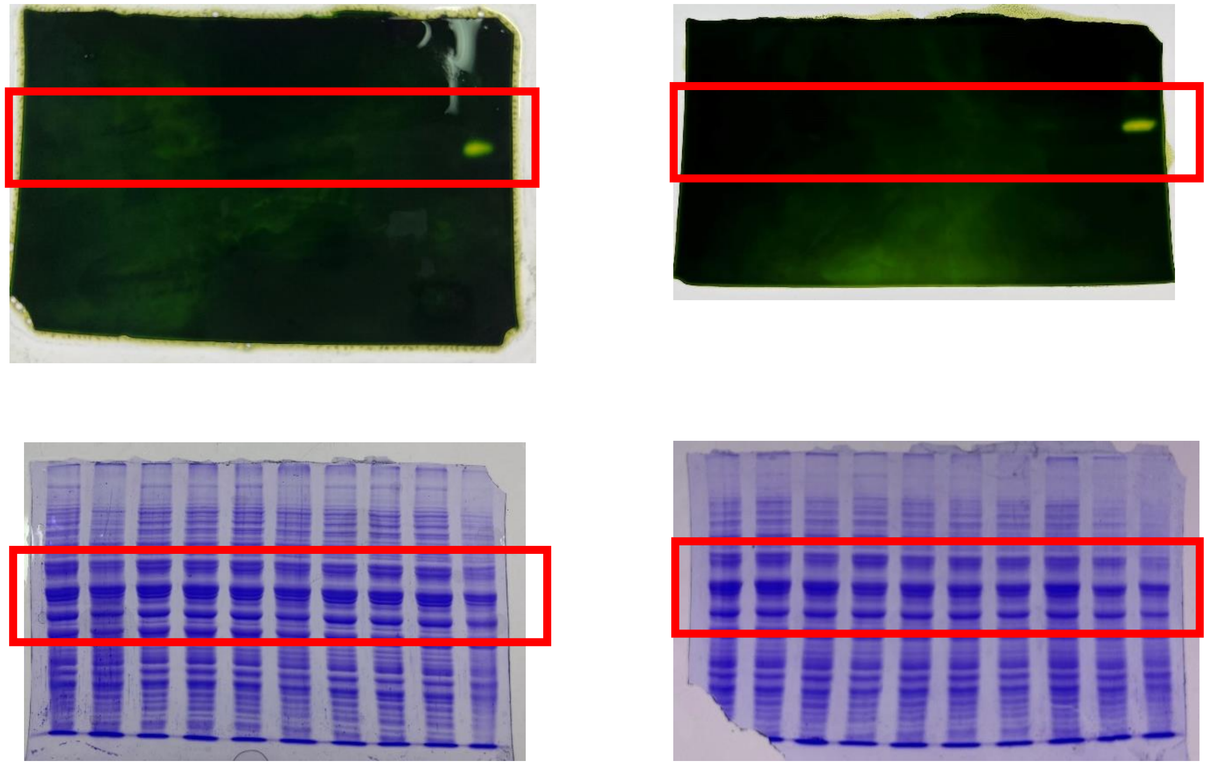

Figure 2B
Bottom panel

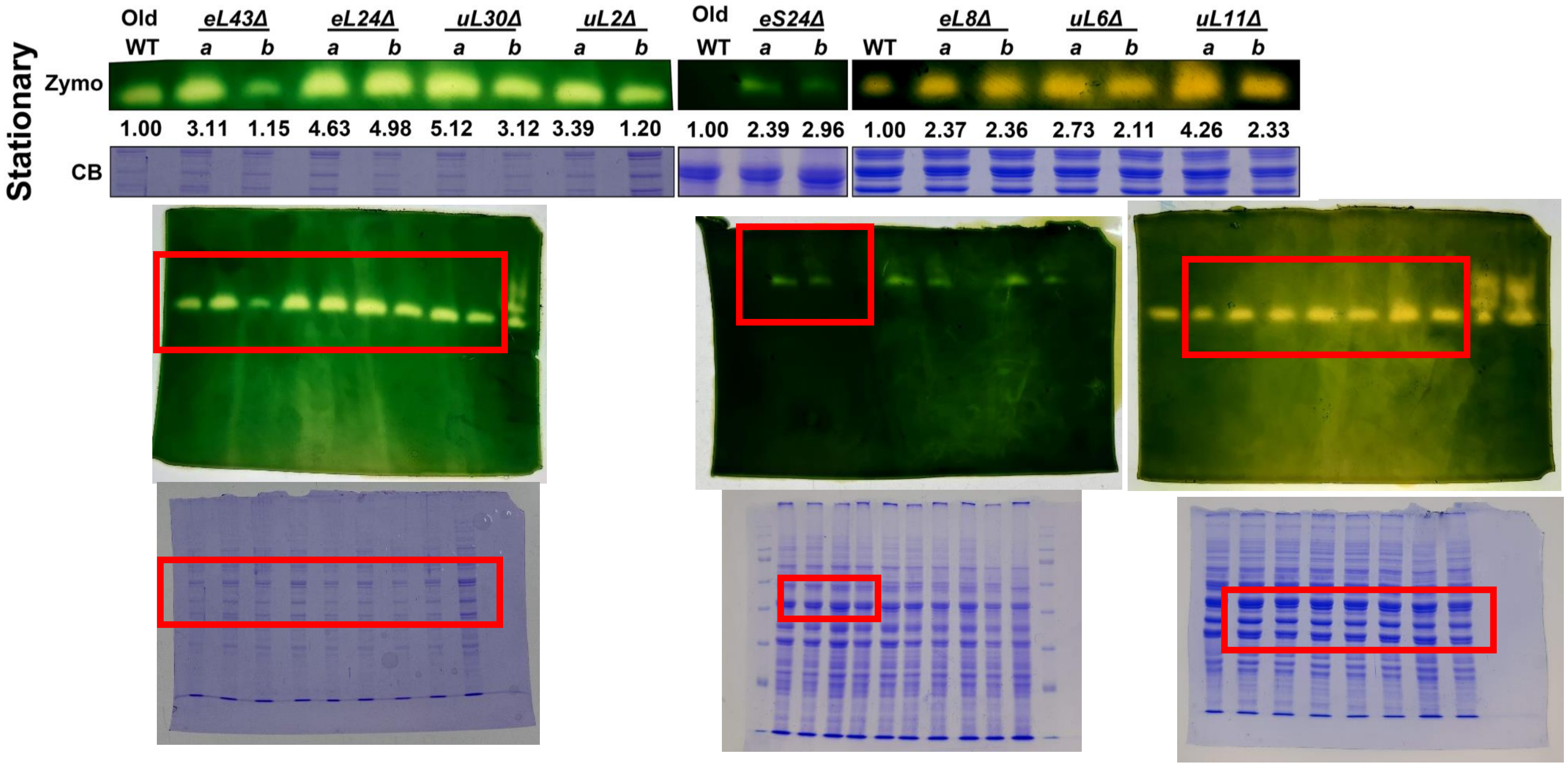

Figure 2C

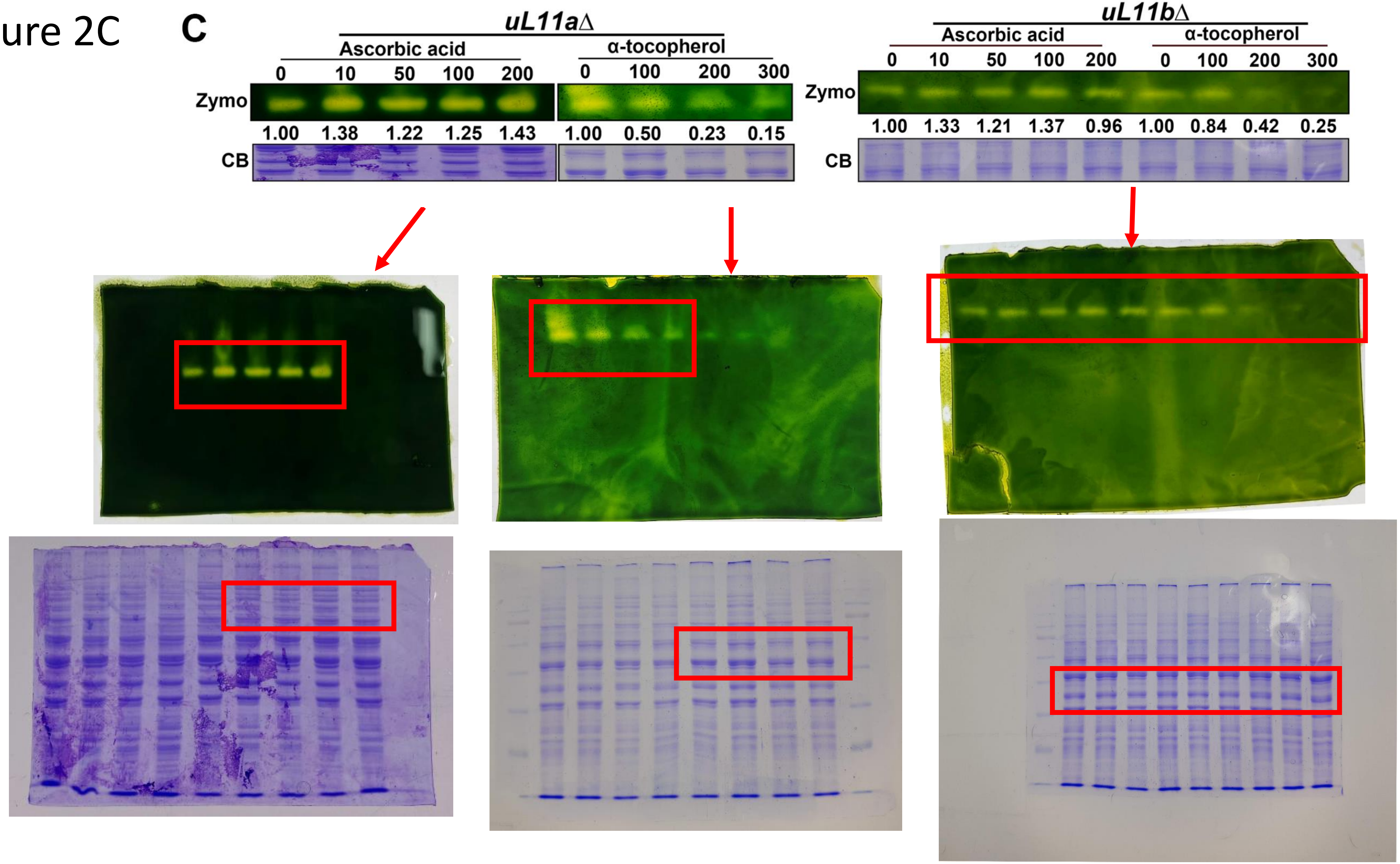
C
uL11aΔ
Ascorbic acid
α-tocopherol
0 10 50 100 200
0 100 200 300
Zymo
1.00 1.38 1.22 1.25 1.43
1.00 0.50 0.23 0.15
CB
uL11bΔ
Ascorbic acid
α-tocopherol
0 10 50 100 200
0 100 200 300
Zymo
1.00 1.33 1.21 1.37 0.96 1.00 0.84 0.42 0.25
CB

Figure 3A

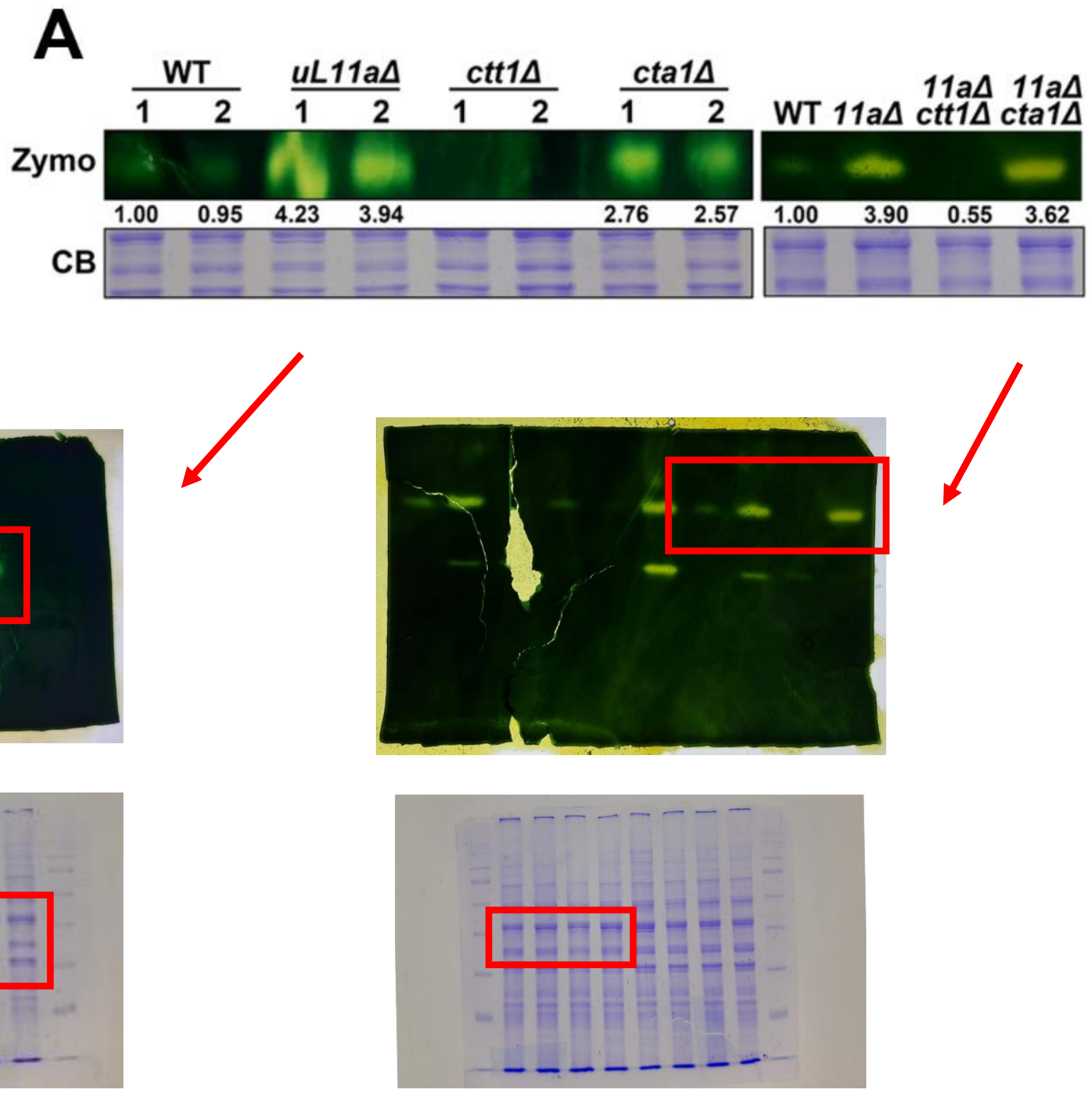
A
WT
uL11aΔ
ctt1Δ
cta1Δ
1 2 1 2 1 2 1 2
WT 11aΔ 11aΔ ctt1Δ 11aΔ cta1Δ
Zymo
1.00 0.95 4.23 3.94 2.76 2.57 1.00 3.90 0.55 3.62
CB

Figure 3B

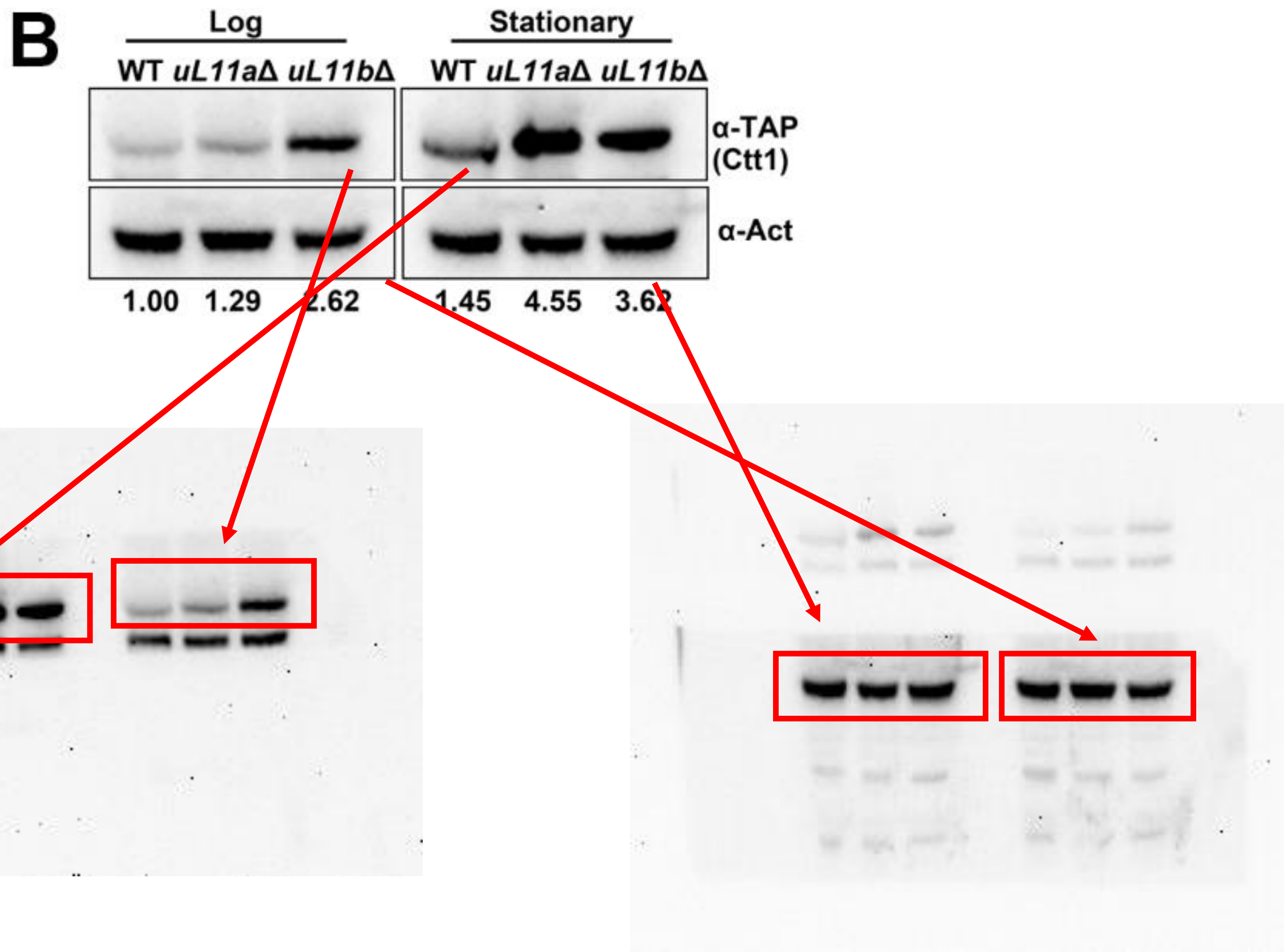
B
Log
Stationary
WT uL11aΔ uL11bΔ
WT uL11aΔ uL11bΔ
α-TAP
(Ctt1)
α-Act
1.00 1.29 2.62
1.45 4.55 3.62

Figure 3B

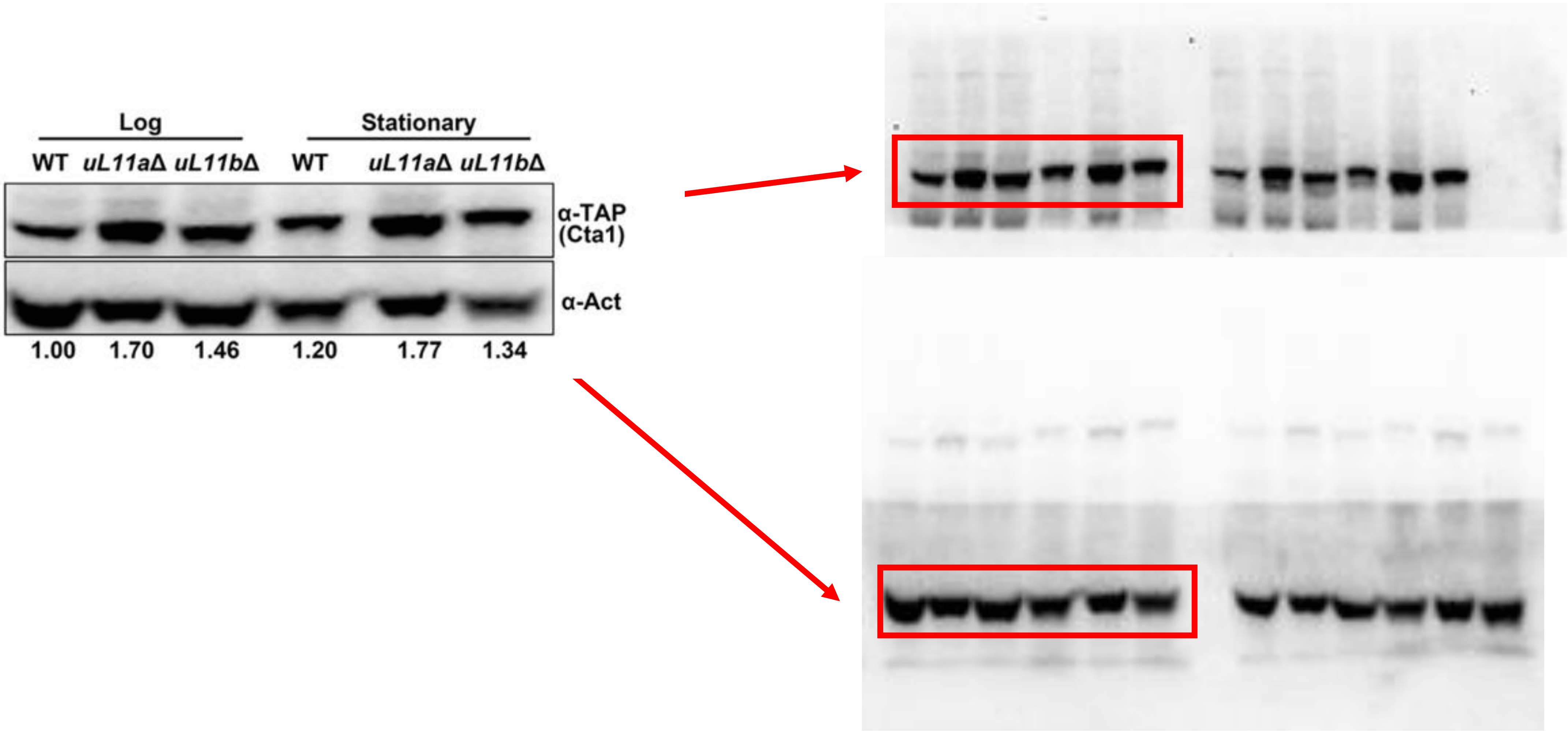
Log
Stationary
WT uL11aΔ uL11bΔ WT uL11aΔ uL11bΔ
α-TAP (Cta1)
α-Act
1.00 1.70 1.46 1.20 1.77 1.34

Figure 3D

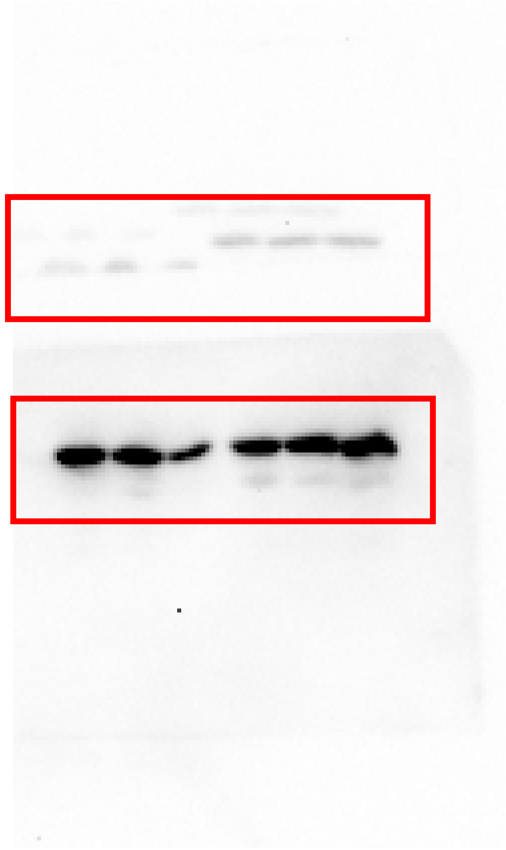

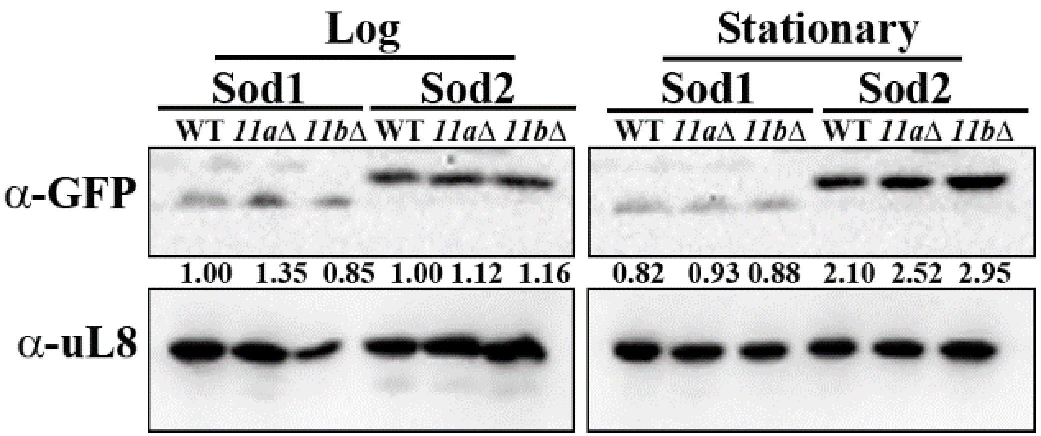
Log
Stationary
Sod1
Sod2
Sod1
Sod2
WT 11aΔ 11bΔ WT 11aΔ 11bΔ
WT 11aΔ 11bΔ WT 11aΔ 11bΔ
α-GFP
1.00 1.35 0.85 1.00 1.12 1.16
0.82 0.93 0.88 2.10 2.52 2.95
α-uL8

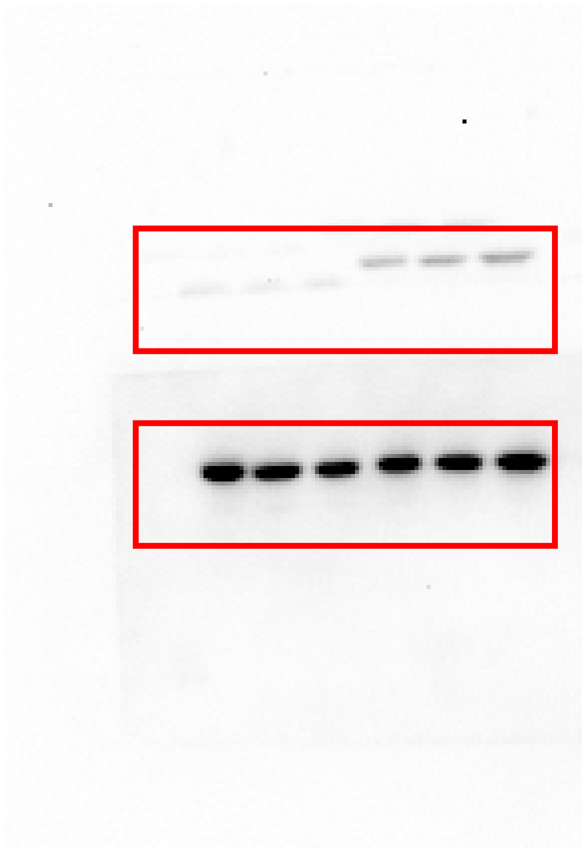

Figure 4A

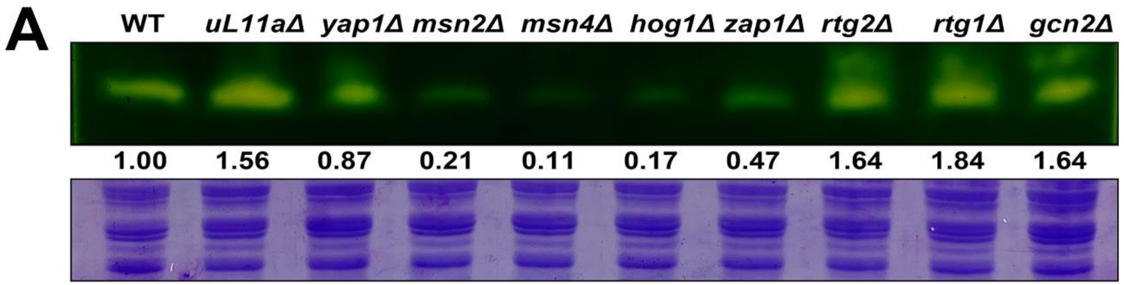
A
WT
uL11aΔ
yap1Δ
msn2Δ
msn4Δ
hog1Δ
zap1Δ
rtg2Δ
rtg1Δ
gcn2Δ
1.00
1.56
0.87
0.21
0.11
0.17
0.47
1.64
1.84
1.64

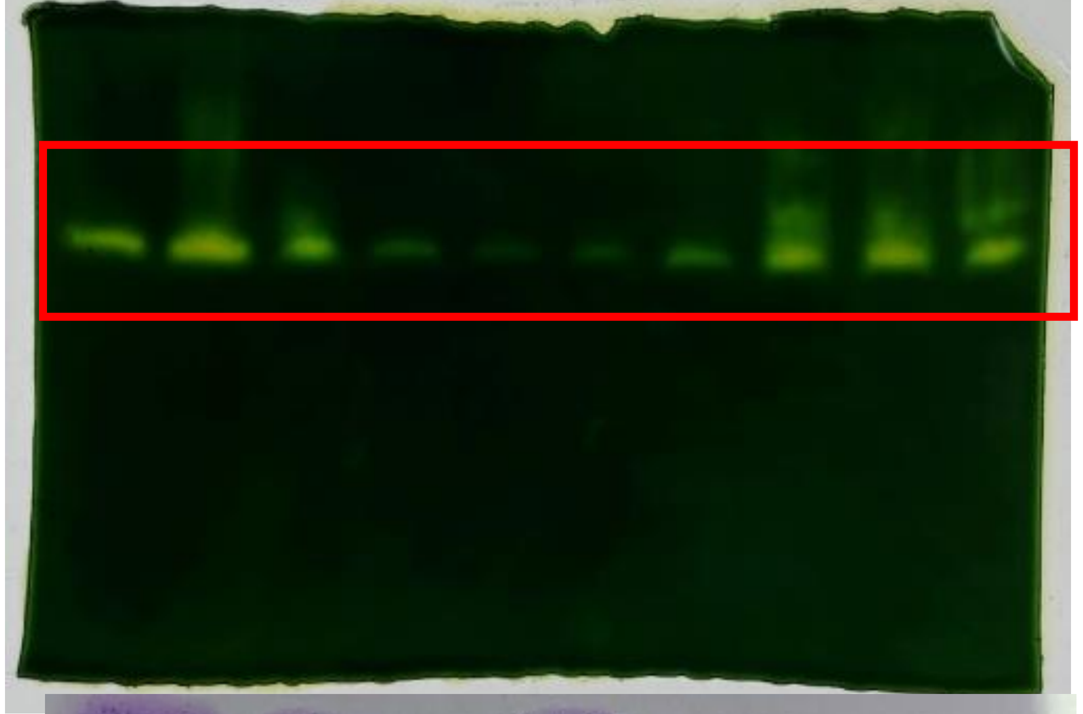

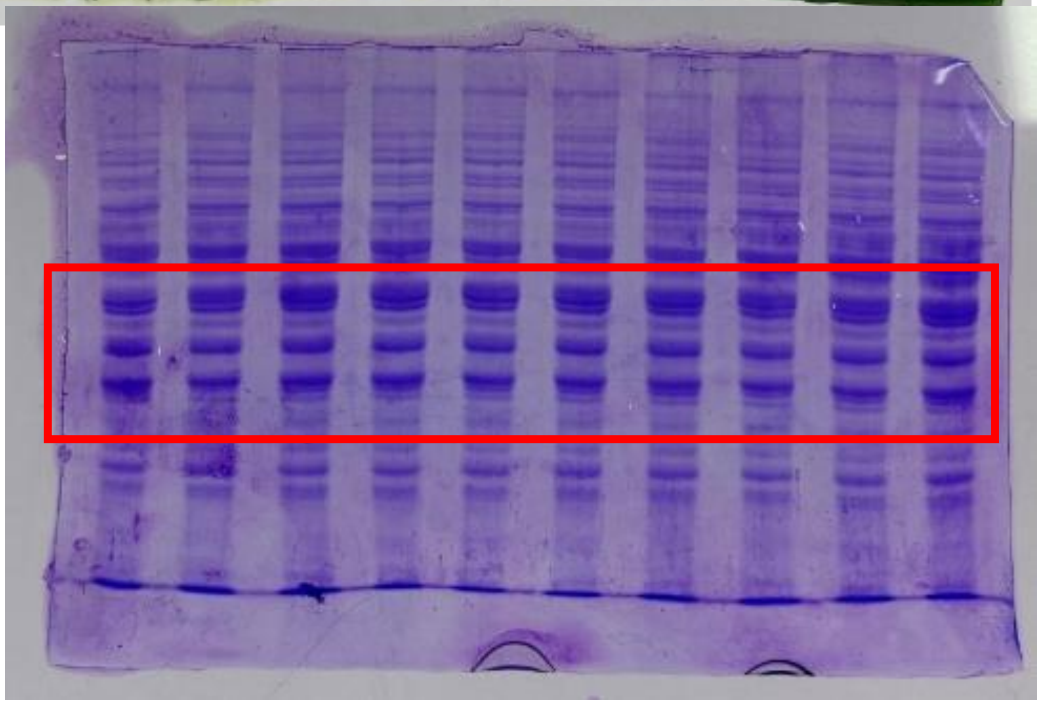

Figure 4C

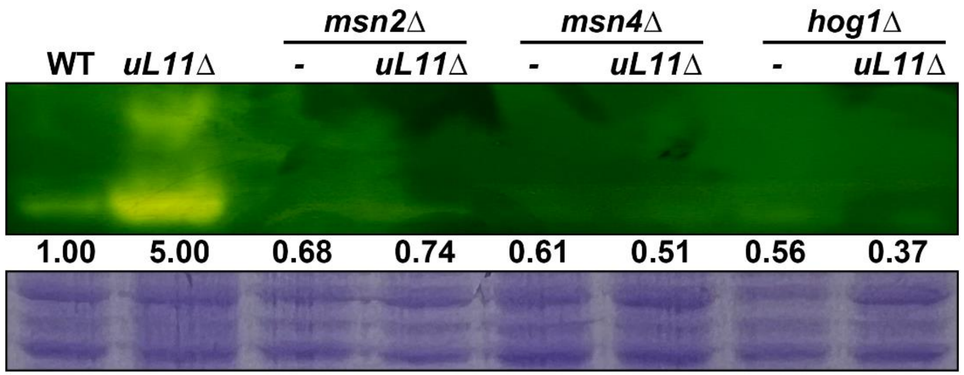
msn2Δ
msn4Δ
hog1Δ
WT
uL11Δ
-
uL11Δ
-
uL11Δ
-
uL11Δ
1.00
5.00
0.68
0.74
0.61
0.51
0.56
0.37

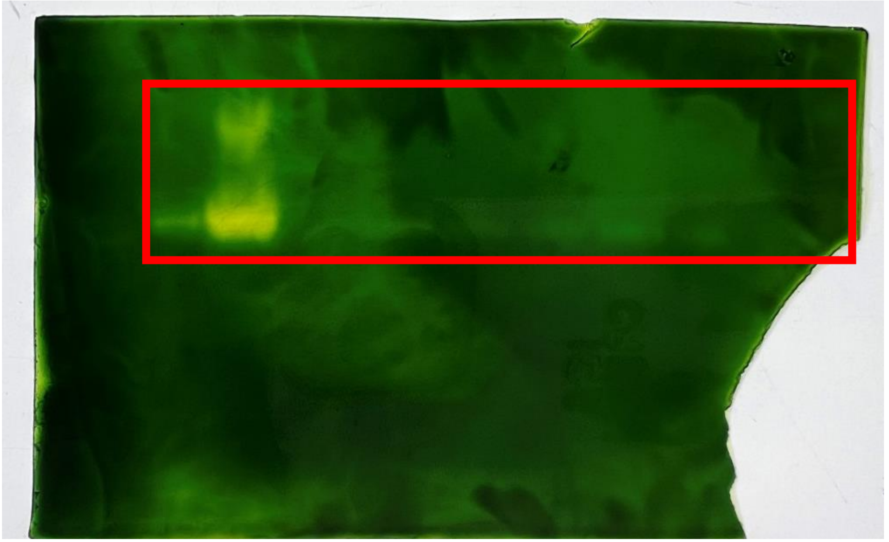

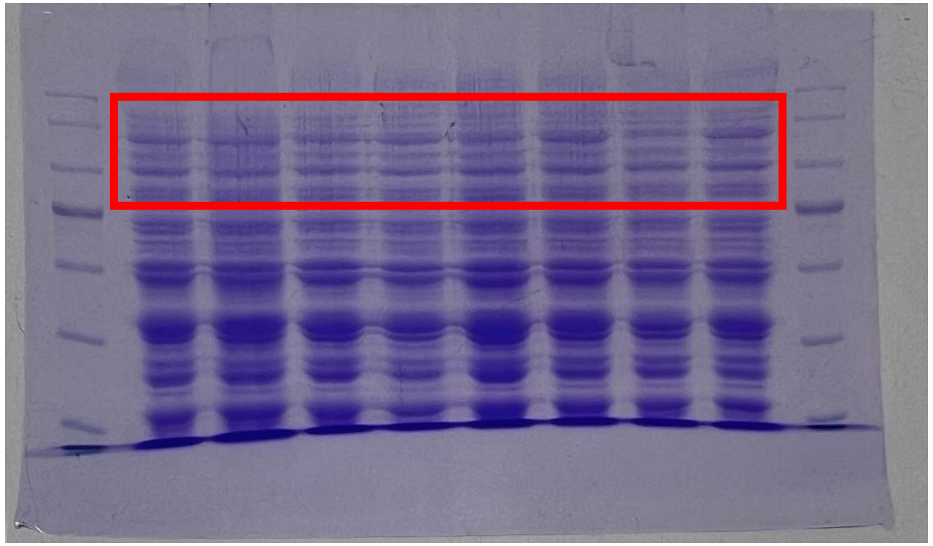

Figure 4D

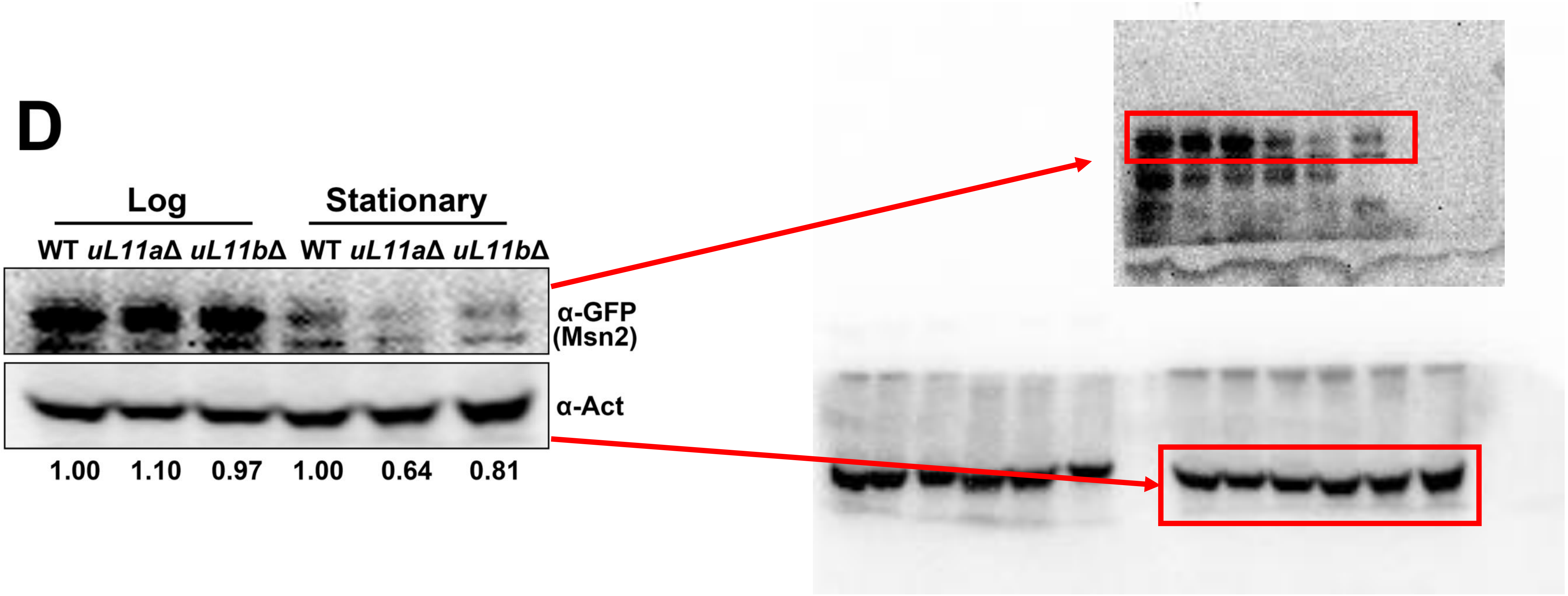
D
Log
Stationary
WT uL11aΔ uL11bΔ WT uL11aΔ uL11bΔ
α-GFP (Msn2)
α-Act
1.00 1.10 0.97 1.00 0.64 0.81

Figure 4D

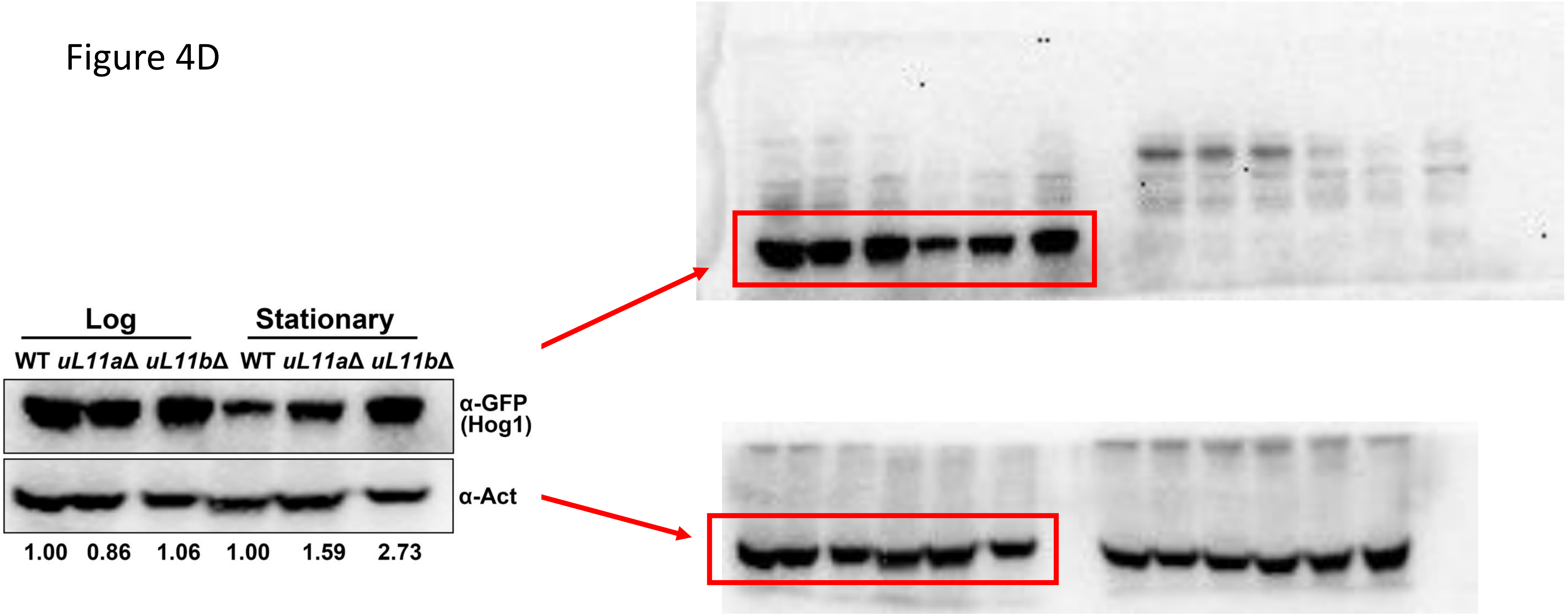

Log
Stationary
WT uL11aΔ uL11bΔ WT uL11aΔ uL11bΔ
α-GFP
(Hog1)
α-Act
1.00 0.86 1.06 1.00 1.59 2.73

Figure 5A

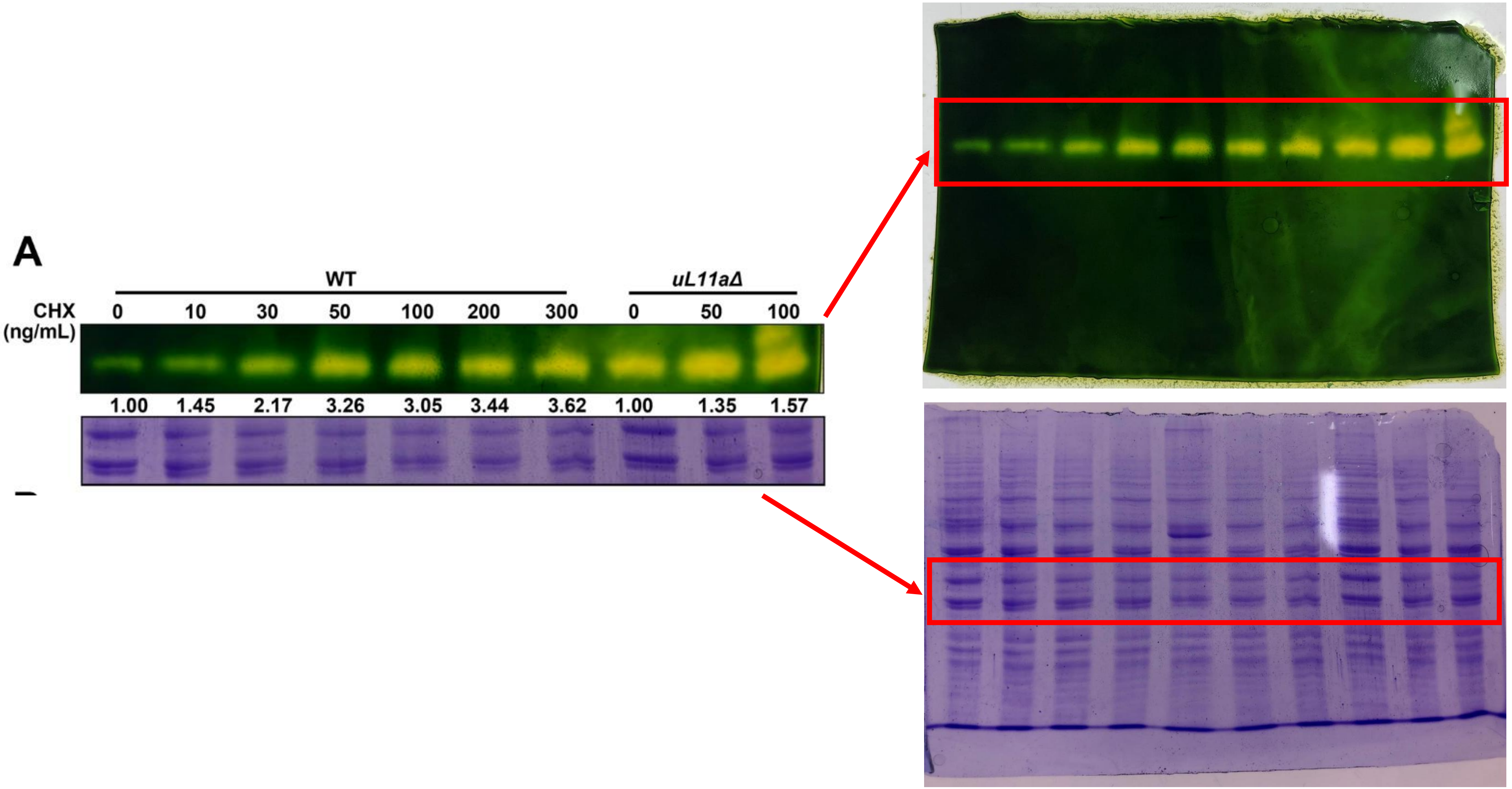
A
WT
uL11aΔ
CHX
(ng/mL)
0 10 30 50 100 200 300 0 50 100
1.00 1.45 2.17 3.26 3.05 3.44 3.62 1.00 1.35 1.57

Figure 5C

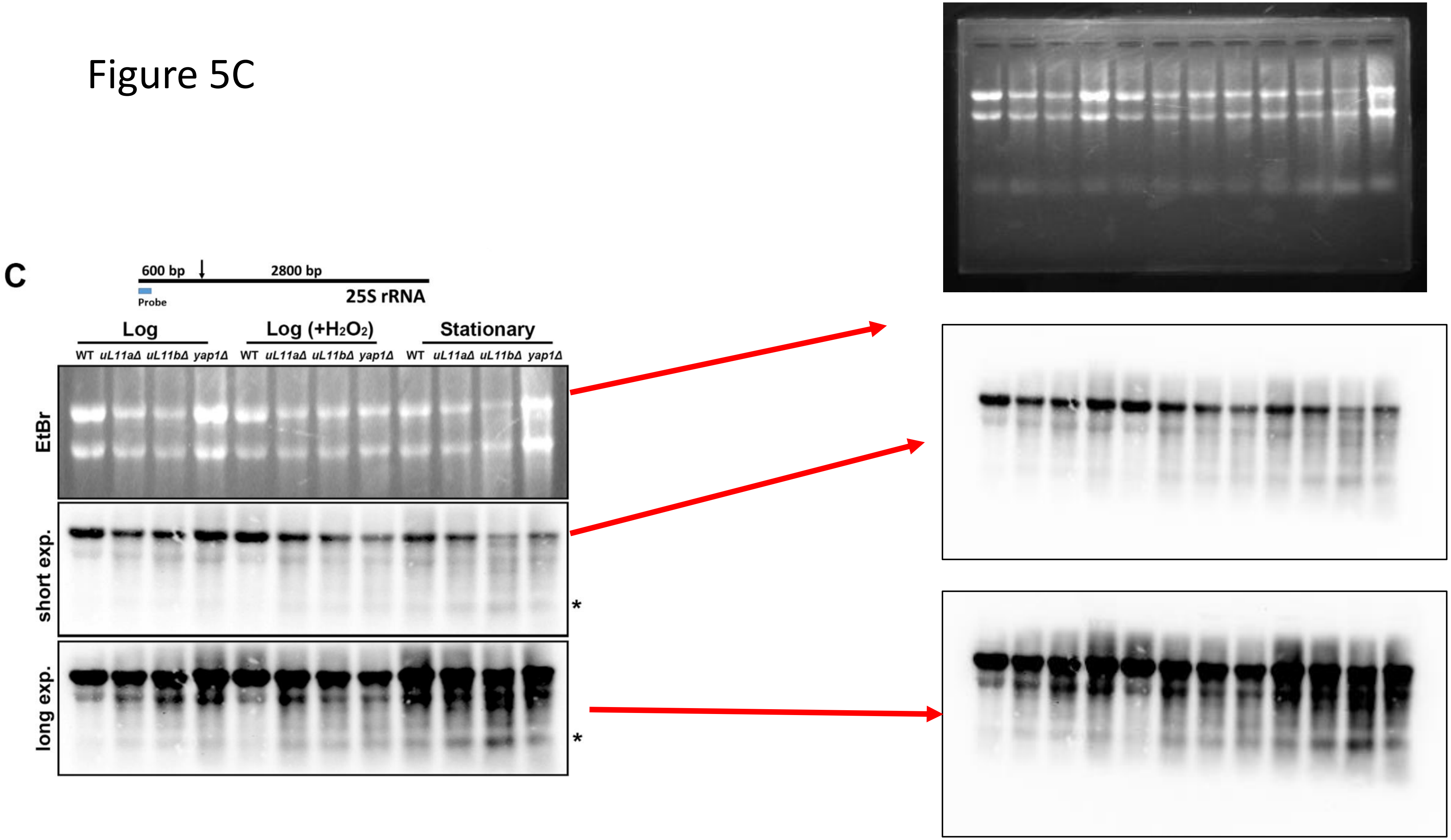
C
600 bp
2800 bp
25S rRNA
Probe
Log
Log (+H2O2)
Stationary
WT uL11aΔ uL11bΔ yap1Δ WT uL11aΔ uL11bΔ yap1Δ WT uL11aΔ uL11bΔ yap1Δ
EtBr
short exp.
long exp.
*
*

Figure S2A

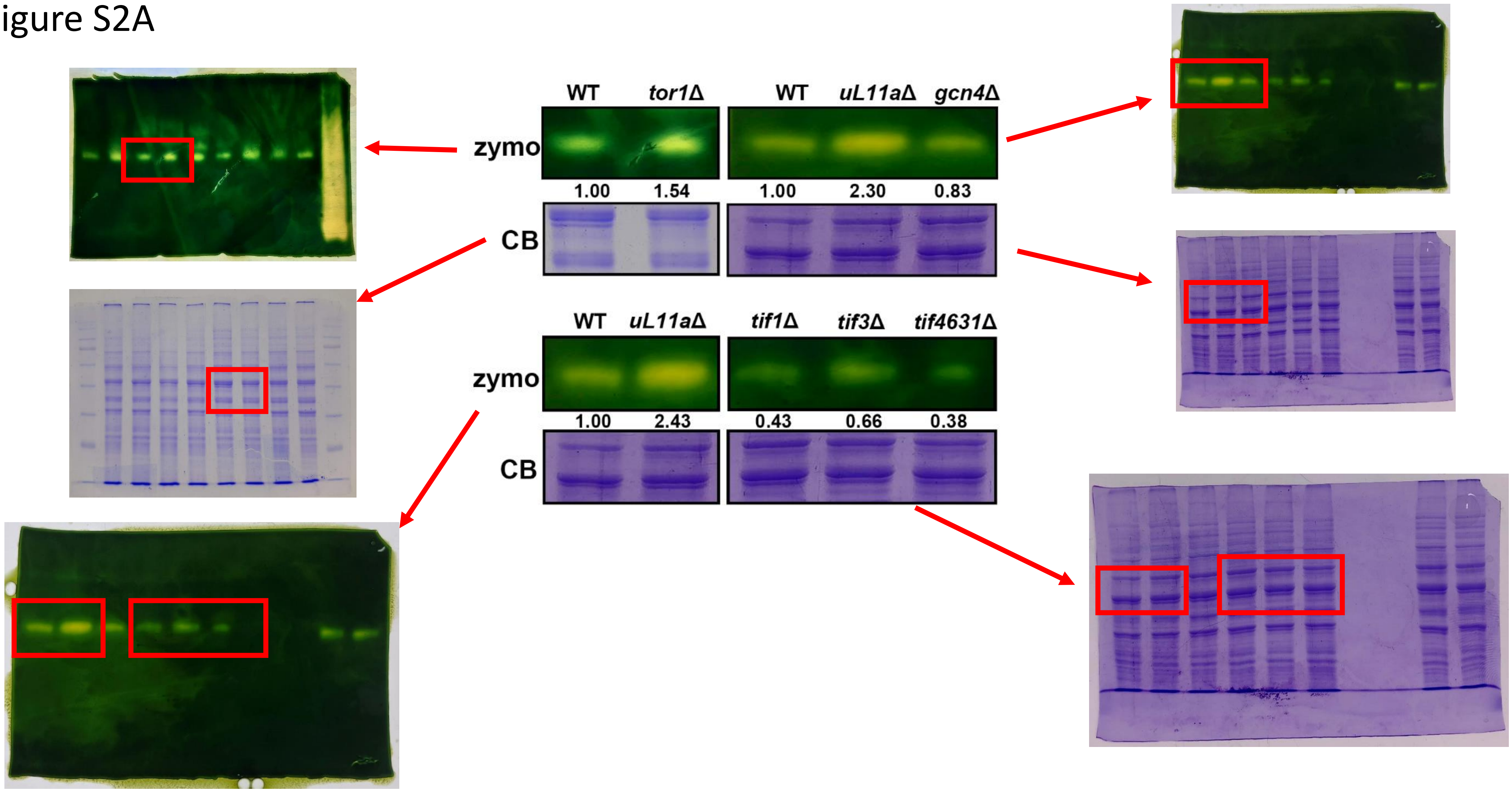
WT
tor1Δ
WT
uL11aΔ
gcn4Δ
zymo
1.00
1.54
1.00
2.30
0.83
CB
WT
uL11aΔ
tif1Δ
tif3Δ
tif4631Δ
zymo
1.00
2.43
0.43
0.66
0.38
CB
